# Utilization of *Pepeta*, a locally processed immature rice-based food product, to promote food security in Tanzania

**DOI:** 10.1371/journal.pone.0247870

**Published:** 2021-03-03

**Authors:** Kulwa F. Miraji, Edoardo Capuano, Vincenzo Fogliano, Henry S. Laswai, Anita R. Linnemann

**Affiliations:** 1 Tanzania Agricultural Research Institute, Ifakara Centre, Ifakara, Tanzania; 2 Food Quality and Design, Wageningen University and Research, Wageningen, The Netherlands; 3 Mwenge Catholic University, Moshi, Tanzania; International Maize and Wheat Improvement center (CIMMYT), MEXICO

## Abstract

Consumption of *pepeta*, a traditionally processed immature rice product, is common in Tanzania, where it contributes to food security as an early rice food i.e. when there is no other food available at the household while the crops in the field not yet fully ripe. Many production steps are needed to guarantee a consistent, good quality *pepeta* product, and this hinders its utilization in other rice-growing sub-Saharan regions. This study aims to gain insight into the *pepeta* processing knowledge and final product, and assess variations in the processing conditions and parameters across the study area. A survey among 257 Tanzanian processors and consumers revealed that the *pepeta* product is widely known, rated second (73.5% respondents) as rice-based food after *wali* (cooked white rice, (100%)) and linked to traditions of the communities in the study area. Harvest of immature rice grain, roasting, pounding, cleaning, and packing are the main process steps of *pepeta* production. Method of rice harvest, rice suitability for *pepeta* production after optimum harvest, dryness of grains and number of pounding as indicator to terminate roasting and pounding process respectively, and packaging materials used varied significantly across respondents in the study area. Reported criteria considered by respondents for product acceptability did not vary significantly across study area. The criteria include colour (76.5%), general appearance (60.8%), texture (64.7%) and taste (52.9%). Immature rice paddy and *pepeta* were sold at a higher price than mature rice paddy and white rice, respectively, which implies that options to facilitate *pepeta* processing through, for instance, standardization of processing conditions and parameters could lead to increased income.

## 1. Introduction

Rice (*Oryza sativa* L.) is an important staple crop for global food security. Its global utilization is estimated at 503.9 million tonnes (milled basis), of which 80.5% in food uses accounting to a per capita food consumption of 53.9 kg [1]. In the sub-Saharan region, including Tanzania, rice farming is a key subsistence activity and serves a dual purpose as a major source of households’ income and food security [2]. In this cropping system, rice is mainly grown on small farms of 0.5–3 ha per household, covering up to 75% of the rice production area [3, 4]. The sub-Saharan region witnesses an increase in rice consumption at a higher pace than ever before due to increased urbanization and an income rise. On average, the annual per capita consumption of milled rice in the region is estimated at 31.0 kg in 2018, which is about 30% more than ten years before [1].

Rice is mainly consumed as milled kernels, i.e. after removing the outer hard layer (husk) to produce brown rice or after further polishing by removing the germ and the inner soft layer (bran) to produce white rice [5]. Nutritionally, the mature dry rice grain contains 80% starch, 12% water, 7.5% protein and 0.5% ash, while providing up to 46 and 43% of dietary energy and dietary protein in the sub-Saharan region, respectively [3, 6]. The rice germ and bran are valuable sources of iron and zinc, dietary fibre, vitamin B (i.e. riboflavin, thiamine and niacin), and vitamin E [3]. The exclusive consumption of white milled rice has caused vitamin and mineral deficiencies, despite the good nutritional potential of whole rice. This is attributed to losses of B vitamins and minerals, which are concentrated in the husk, germ and bran, which are removed during milling [7].

Studies to improve the nutritional quality of milled rice generally focus on optimising product and processing aspects. These include rice flour processing and development of related products such as fermented, baked, extruded and fried products [8]. Hydrothermal rice processing technology, i.e., parboiling significant improve the nutritional quality of rice by enhancing the diffusion of some minerals and water soluble vitamins into the endosperm [9, 10]. Most studies concern mature rice, with little attention on the use of immature rice grains. Use of immature cereal grains has potential nutritional benefits, since nutrient contents tend to decrease as grain matures. Several studies reported higher amounts of nutritive components such us protein, reducing sugar, calcium, potassium, iron, β-carotene, vitamin C, vitamin B2, B3 and B6, vitamin E and γ-oryzanol in immature grains when compared to mature grains [11–13].

Physical and economic access to nutritious food is among main components of food security [14]. Sales of crops to meet household cash obligations have been linked to the improvement of sustainable food and livelihood security [2, 15]. Apart from premium selling, use of immature grains can directly increase smallholder farmers’ income through reduced farm management costs due to early harvest and exclusion of postharvest operations like drying and storage. The gained cash income can either be used by households to increase yields (hence physical food accessibility) through improved crop management practices or obtain food from the local market (economic food accessibility) [2].

Currently, there is a growing interest at valorising potentially nutritious though neglected immature cereal-based food products [16–18]. Traditional processing of immature rice to produce *pepeta* is common in Tanzania and has existed in isolation from rest of the sub-Saharan region. *Pepeta* is widely consumed by communities because its natural flavour, which resembles the buttered popcorn aroma. As snack food, *pepeta* has potential due to increased consumer’s demand for nutritious, healthy, ready-to-eat processed food with satisfying taste and ease of portability because of rising urbanization and increased employment of women in industrial and public sectors worldwide [19, 20]. However, very little information (except nutrition composition [21]) is available in the literature related to *pepeta* processing knowledge and the final product. Therefore, the present study was undertaken to gain insight into the *pepeta* processing knowledge and assess variations in the processing conditions and parameters across the study area. Perceived *pepeta* product quality characteristics, its role in the community including history, traditions, taboos and uses, and *pepeta* problems and trade supply chain in the study area were investigated as well. The documented information serves as a basis for research into possible ways to optimise specific processing conditions to improve nutritional quality, and/or other fields along *pepeta* value chain to enhance its competitiveness.

## 2. Methodology

### 2.1 Study design

Both quantitative and qualitative approaches were used in this study. A face-to-face interview through questionnaires together with focus group discussion and on-field observation via demonstrations were the main data collection tools used. The questionnaire was based on a conceptual model that represent simplified *pepeta* value chain and its link to food and livelihood security.

### 2.2 Questionnaire design

#### 2.2.1 Conceptual model used to design the questionnaire

**Fig 1:**
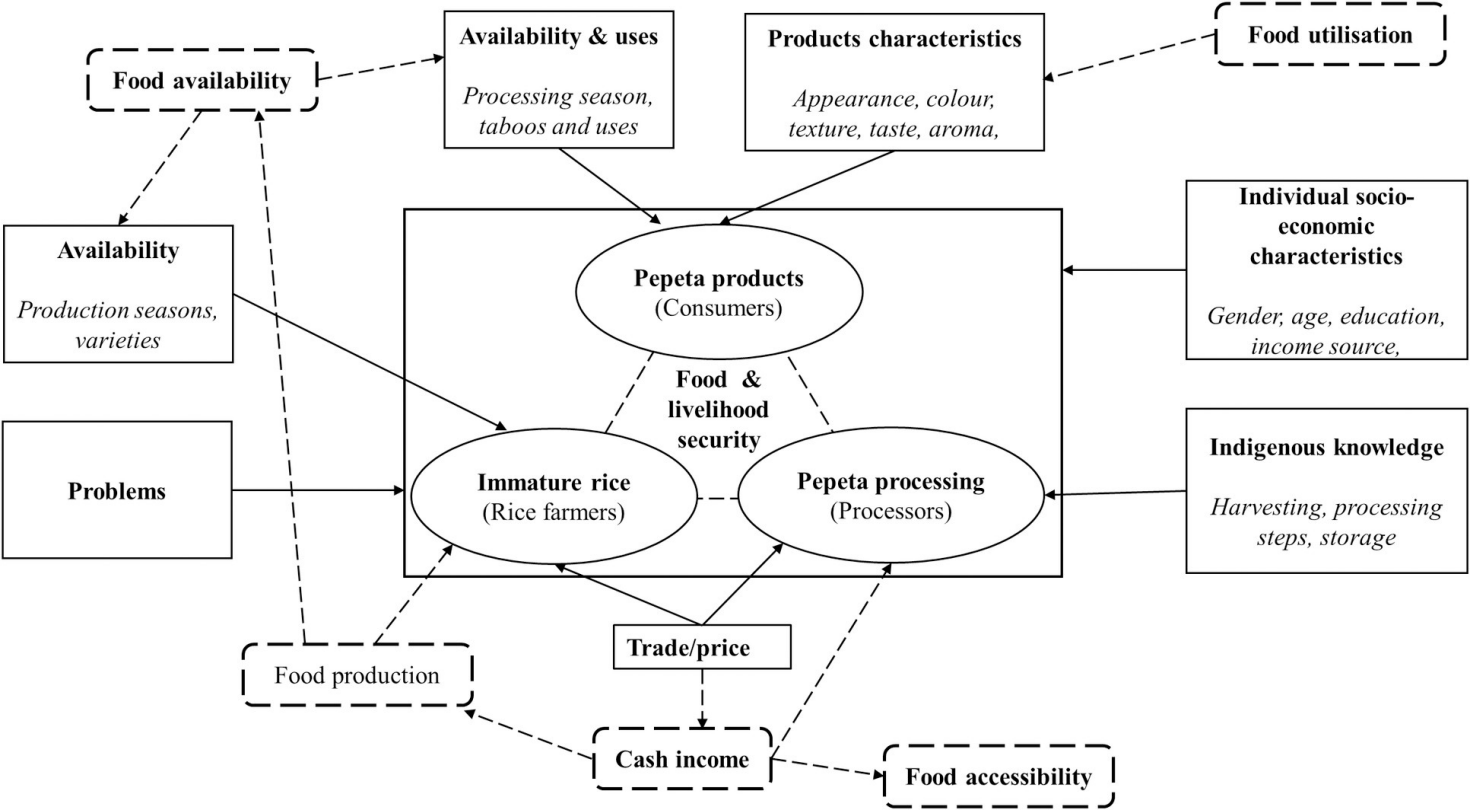
Conceptual framework for the design of the questionnaire used to collect information along *pepeta* value chain. _______ indicates possible factors that can affect *pepeta* value chain, and _ _ _ _ indicate elements of *pepeta* processing interact with food and livelihood security.

Fig 1 show a conceptual model used to design the questionnaire (S1 Questionnaire) and checklist (S1 Checklist). The model reflects the key actors (rice farmers, processors and consumers) along *pepeta* value chain and its linkage to food and livelihood security. Immature rice grains are sole ingredients for processing of *pepeta* products, the availability and accessibility can substantially affect *pepeta* processing and consumption. Rice is preferred for human consumption and plays a significant role in Tanzanians’ culture, traditions, and religion [22]. It is a major source of income, food and employment in rural areas, providing about 95% of the national food requirements, accounts for more than 70% livelihoods of the Tanzanian population [23]. The influence of processing conditions on product quality properties and acceptability are fundamental in processed food products like *pepeta* [24], affecting food product utilization. Therefore, indigenous knowledge on immature rice grains harvesting and processing, information on *pepeta* product quality and acceptability, and existing problems are included in the model as well. Food product characteristics such as appearance, taste, texture, colour, aroma, and socio-economic characteristics of consumer like gender, age, education and income can influence food choices and preference [25].

#### 2.2.2 Questionnaire

The questionnaire was developed based on the conceptual model (Fig 1) to collect information on the following aspects: (1) General information and awareness of *pepeta*, (2) *Pepeta* processing knowledge, (3) Product quality criteria and utilization, and (4) Product processing, marketing and storage constraints. It was divided into sections containing specific questions to extract information from different actors along *pepeta* processing value chain (S1 Questionnaire), as follows:

- *General information*: Gender, age, education, income source, awareness about *pepeta* and type of actor in the value chain, i.e., processor, consumer and/or trader.
- *At consumer level*: Consumer information on consumption frequency of *pepeta*. Place of purchase (e.g., informal market, farmer-gate, and/or supermarket), prices relative to product quality (freshness and seasonality). Properties considered during purchase (e.g. colour, appearance, texture, taste and aroma).
- *At processor level*: *Pepeta* processing techniques and product quality. Sources of raw materials (immature paddy), parameters considered when purchasing raw material for *pepeta* processing (i.e., varieties and maturity level) and prices. Places of sales of *pepeta*, consumer perception of product quality (e.g., colour, appearance, texture, taste and aroma), constraints and most tedious process steps.
- *At trade level*: Places of sales of *pepeta*, consumer perception of product quality (e.g. colour, appearance, texture, taste and aroma).

### 2.3 Study area

**Fig 2:**
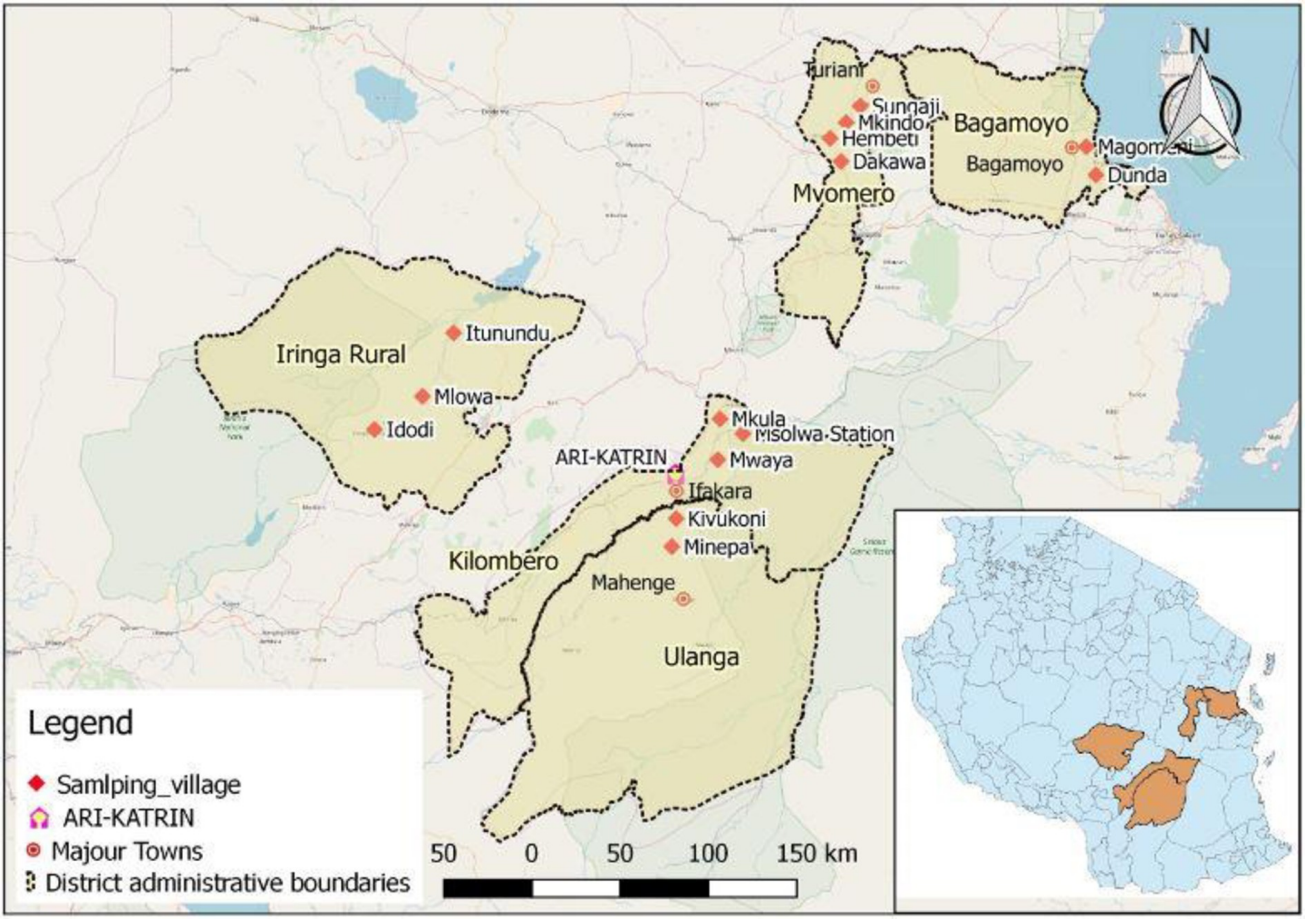
Study area surveyed for *pepeta* product and its indigenous processing knowledge in Tanzania. Coordinates for district administrative boundaries are indicated as follows: Kilombero (08^o^07’S, 36^o^40’E), Ulanga (09^o^00’S, 36^o^40’E), Mvomero (06^o^18’S, 37^o^27’E), Bagamoyo (06^o^26’S, 38^o^54’E) and Iringa rural (07^o^46’S, 35^o^42’E). ARI-KATRIN = Agricultural Research Institute KATRIN. Source: Survey data, September 2017 –February 2018. The map background and shapefiles were taken from OpenStreetMap Foundation (OSMF, United Kingdom) and National Bureau of Statistics (NBS, Tanzania) with permission under a CC BY license, respectively.

The study was conducted in the Eastern and Southern Highland parts of Tanzania, focusing on five district administrative boundaries: Kilombero (08^o^07’S, 36^o^40’E), Ulanga (09^o^00’S, 36^o^40’E), Mvomero (06^o^18’S, 37^o^27’E), Bagamoyo (06^o^26’S, 38^o^54’E) and Iringa rural (07^o^46’S, 35^o^42’E) (Fig 2). The study area comprises several valleys that are considered among the most fertile areas in the country, suitable for irrigation agriculture, including rice crop cultivation. The valleys include Great Ruaha River (208 000 ha) and the Little Ruaha floodplain (4800 ha) in Iringa rural district council, the Kilombero valley floodplain (329 600 ha) in Kilombero and Ulanga district councils, the Wami floodplain (169 000 ha) in Mvomero district council, and the Ruvu floodplain (117 000 ha) in Bagamoyo district council [26] The district administrative areas were chosen based on the relevance of rice as one of key crops in the farming system and its dual purpose as a major source of households’ income and food security. According to the perception of local people (community leaders) and Village Agriculture and Extension Officers (VAEOs), the area has also been serving as a centre of *pepeta* processing knowledge for many decades.

### 2.4 Sampling of respondents/informants

Prior to data collection, a random observation was done on 117 food venders selling food at local markets to estimate the proportion “p” of venders selling rice-based food products. The research team assessed whether a vender was selling at least one common rice-based food product found in the community. The obtained proportion was then used to estimate the total number “n” of respondents to be interviewed in the study, using the following formula [27]:

$$n=\frac{{U_{1}^{2}}_{-\propto /2}\times p(1-p)}{d^{2}}$$

where n is the total sample size; p is the proportion of targeted informants; *U*_1−∝/2_ = value of the Normal random variable for a probability value of 0.975 (or α = 0.05), *U*_1−∝/2_≈1.96; d = margin error of the estimation fixed at 5% (0.05).

The district population size was used to compute the number of participants to be interviewed for each district council (Table 1) according to [28]:

$$ni=\frac{Ni}{N}*n$$

where n_i_ is the number of participants in the district council; N_i_ is the total number of the population in the district council; and N is the total number of people living in the five selected district councils. The used population data were based on Tanzania National Bureau of Statistics report [29].

**Table 1:** Population distribution overview in the study area.

District council	Population size*	Proportion of the population	Number of informants
Kilombero	339,092	0.27	69
Ulanga	169,853	0.14	36
Mvomero	351,075	0.28	71
Bagamoyo	108,811	0.09	23
Iringa rural	268,840	0.22	56
Total	1,237,671	1	255

*According to Tanzania National Bureau of Statistics (NBS), [29].

### 2.5 Field data collection

Data collection was from September 2017 to February 2018, using a structured questionnaire, by focus group discussions and observations. The questionnaire was first tested and adjusted before being administered to the 257 respondents selected from five district administrative boundaries (Table 2). For face-to-face interviews, respondents from each household in the selected villages were randomly selected from the list of rice farmers and/or *pepeta* processors who were willing to participate provided by VAEOs. The interviews were held in *Swahili*, the national language of Tanzania, which was well understood by all respondents.

**Table 2:** Demographic profile of respondents (as percentages) in the surveyed administrative districts for *pepeta* product and its indigenous processing knowledge in Tanzania.

	Districts					Overall % (n = 257)
	Kilombero (n = 68)	Ulanga (n = 38)	Mvomero (n = 72)	Bagamoyo (n = 24)	Iringa rural (n = 55)	
Gender*						
Males	26.5	15.8	40.3	70.8	67.3	41.6
Females	73.5	84.2	59.7	29.2	32.7	58.4
Age group (years)*						
18–29	20.6	23.7	19.4	8.3	20.0	19.5
30–44	47.1	39.5	33.3	8.3	40.0	37.0
45–59	27.9	31.6	34.7	50.0	29.1	32.7
60^+^	4.4	5.3	12.6	33.4	10.9	10.9
Level of education*						
No education	13.2	15.8	9.7	8.3	0.0	9.3
Primary level	79.4	78.9	73.6	62.5	78.2	75.9
Others ^a^	7.4	5.3	16.7	29.2	21.8	14.8
Main source of livelihood ^b^						
Agricultural activities*	52.9	26.3	66.7	54.2	70.9	56.8
Rice cultivation only*	48.5	73.7	33.3	45.8	29.1	43.6
Pepeta processing*	50.0	52.6	0.0	0.0	0.0	21.0
Others*^c^	1.5	7.9	41.7	45.8	49.1	28.0

^a^ Combined secondary level, vocational training and tertiary/collage options since the number of each case was small

^b^ more than one answer possible

^c^ combined casual labour, remittances, petty trade, mechanics and civil servant options since the number of each case was small

*Significance difference among districts at p < 0.05.

Source: Survey data, September 2017 –February 2018.

Focus group discussions were employed to comprehend the collected information from individual face-to-face interviews, as people normally tend to mention the most important things when asked to freely recall under a given short time [27]. Such discussions consisted of 12–15 participants, purposively selected following criteria such as age (youth, people of reproductive age and old), gender balance, and different actors (farmers/consumers, processors and traders) along the *pepeta* processing value chain (Table 3). The focus group discussions last for 2–3 hours for each conversation and were guided by a checklist. A total of 5 focus group discussions were conducted; 3 in Kilombero district and 2 in Ulanga district, depending on the population sample size. The observation data were collected during harvesting and handling of rice and *pepeta* processing to verify data collected from interviews. Physical properties data i.e. moisture content, weight, temperature and duration of various processing steps of *pepeta* production were measured using a digital grain moisture meter (SATAKE, MOISTEX SS7), weighing scale (Endel™, EWS-H-PLUS), digital thermometer (Fluke, model 52 II), and digital timer (Fisherbrand™), respectively.

**Table 3:** Overview of focus group discussion.

Participants category	Number of participants	
	Kilombero district	Ulanga district
Gender		
Male	6	7
Female	6	8
Total (per FGD*)	12	15
Age		
Youth (< 30 years)	2	2
Adults (30–49 years)	4	5
Elders (> 49 years)	6	8
Total (per FGD)	12	15
*Pepeta* actors		
Rice farmers/consumers	6	8
*Pepeta* processors/traders	6	7
Total (per FGD)	12	15
Number of FGD conducted	3	2

*Focus group discussion. Source: Survey data, September 2017 –February 2018.

The study was approved by and conducted in collaboration with Tanzania Agricultural Research Institute (TARI)–Ifakara center, following all relevant regulations. The individual pictured in Figs 5 and 6 has provided written informed consent (as outlined in PLOS consent form) to publish their image alongside the manuscript.

### 2.6 Data processing and analysis

Data from individual household interviews were subjected to descriptive statistics (percentages or frequencies) using IBM SPSS statistics (version 23, USA) and Microsoft Excel 2016. A chi-square test of independence at 0.05 level of significance was performed to determine if there was significant different between *pepeta* processing practices of Kilombero and Ulanga respondents and ascertain the relationship between *pepeta* knowledge (awareness) and respondents’ demographic data (gender and age). The chi-square test was performed independently for each individual option in the multiple response questions. Data for process efficiency, and moisture, weight and heat losses, the independent t-test (p < 0.05) was computed to evaluate any significant different between Kilombero and Ulanga districts. Qualitative data gathered through focus group discussion and observation were content analysed. The collected GPS data were used to prepare a map using QGIS (version 3.4.3).

## 3. Results and discussion

### 3.1 Product history, taboo and uses

According to the focus group discussions, *pepeta* means *“flattened grains”* and its processing knowledge dates back since introduction of rice in the community. This knowledge has been passed on from generation to generation. *Pepeta* and its processing knowledge form an integral part of social rites, rituals and festivals of the *Ndamba*, *Mbunga*, *Ngindo*, *Pogoro*, *Kwere* and *Doe* ethnic tribes found in the study areas as mentioned by respondents. According to the interviewees, no girl would qualify for marriage if she would not know how to process *pepeta*. Therefore, *pepeta* processing was one of the trainings given to young girls during *unyago* rituals, a practice to celebrate the coming of age of girls or during weddings. According to *kwere* and *doe* tribes, a grounded *pepeta* product, known as *bwimbwi*, was used as a participation fee in *jando*, a circumcision rite for boys. *Unyago* and *jando* rituals involved instructing youth about sex and conjugal life [30]. It was further believed that a marriage became happy if a spouse (wife) prepared a large amount of *pepeta* to proudly serve her husband throughout a year or until the next rice harvesting season. Preparing *pepeta* is a way of showing affection and therefore *pepeta* was also given to special guests like in-laws as a symbol of respect and care. Reserving traditional snacks for special, esteemed individuals like men and in-laws is a common practice among Tanzania communities [31].

*Pepeta* was also recognized as a symbol for the start of a new harvest season for *Ndamba*, *Mbunga*, *Ngindo* and *Pogoro* tribes. No household was allowed to start the new harvest before *pepeta* was prepared and sent to the *chief*, a community leader, for making a ritual sacrifice asking protection against natural calamities, wild animals and birds.

In the study area, respondents explained their reason for using immature rice to prepare *pepeta*: as a means of securing food when no other food was available at the household while the crops in the field not yet fully ripe. Therefore, mothers were forced to prepare *pepeta* to feed their children while waiting for the main dish. These findings illustrate the tremendous importance of *pepeta* in the community living in the surveyed areas: “a means of communication, affirming and reinforcing social relations, of expressing one’s personal or group identity and of connecting to the living or ancestral peer group [32]”.

### 3.2 Product popularity

Though the popularity of *pepeta* varied significantly (*x*^2^ = 109.193, df = 4, p = 0.000) across respondents in the study areas, most of the respondents (73.5%) knew the product and rated the product second after *wali* (cooked white rice) (Table 4), the main form of consuming rice in Tanzania [23]. *Pepeta* was mentioned as one of the common rice-based food products by 100% of respondents in Kilombero and Ulanga districts, and by 79.2 and 70.8% in Bagamoyo and Mvomero rural districts, respectively. Contrarily, only 23.6% of respondents from Iringa rural districts knew *pepeta*. This difference could be due to observation that *pepeta* was no longer processed (hence unavailable) in Iringa rural district, whereas it is regularly processed for both sales and household consumption in Kilombero and Ulanga districts, and occasionally processed in small quantity for household consumption in Mvomero and Bagamoyo districts. *Pepeta* processing, as traditional knowledge, is attributed to several factors including gender and generation [32]. To confirm this concept, the association between *pepeta* knowledge (awareness) and respondents’ demographic data (gender and age) among districts (Mvomero, Bagamoyo and Iringa rural) was tested. The analysis in Table 4 show gender differed significantly (*x*^2^ = 8.817, df = 2, p = 0.012) across respondents in the districts, more males knew *pepeta* product in Bagamoyo and Iringa rural districts, and the vice versa is true for Mvomero district. However, no significant differences were found in the overall respondents between males (53.0%) and females (47.0%) for *pepeta* knowledge (*x*^2^ = 3.417, df = 1, p = 0.065), indicating location is the main influencing factor for the observed differences across districts in the study area. To evaluate the impact of age on *pepeta* knowledge, respondents were categorized into three groups: below 30 years (regarded as youth with little or no knowledge of *pepeta*), between 30 and 44 years (regarded as adults with moderate *pepeta* knowledge), and 45 years and above (considered as elders with much experience on *pepeta*). There was a significant difference in the overall respondents regarding *pepeta* knowledge among age groups (*x*^2^ = 9.696, df = 2, p = 0.021), with 61.5% of those familiar with *pepeta* (55% of the respondents) being 45 years and above, while 12% were below 30 years (Table 4). Also, age groups differ significantly (*x*^2^ = 6.265, df = 4, p = 0.018) across the respondents in the study area. The data suggest that *pepeta* processing, like other traditional knowledge, is at risk of extinction due to adaptation to surroundings and culture changes from one generation to another [32]. In this study no data about *pepeta* processing and/or consumption was collected at Mvomero, Bagamoyo and Iringa rural districts as respondents in these districts could not recall the last time they processed and/or ate *pepeta*. They obtained *pepeta* from local markets or as a gift from Kilombero and Ulanga folk.

**Table 4:** Frequencies (as percentage) of respondents on awareness of rice-based products and *pepeta* knowledge in the study area.

	Districts					Overall (%)
	Kilombero	Ulanga	Mvomero	Bagamoyo	Iringa rural	
Rice-based product popularity ^a^	n = 68	n = 38	n = 72	n = 24	n = 55	n = 257
Pepeta*	100.0	100.0	79.2	70.8	23.6	73.5
Wali (cooked white rice)^#^	100.0	100.0	100.0	100.0	100.0	100.0
Wali (cooked brown rice)*	23.5	0.0	18.1	0.0	1.8	11.7
Mchopeko (parboiled rice)*	48.5	31.6	15.3	0.0	0.0	21.8
Vitumbua (rice dough)*	66.2	71.1	70.8	45.8	87.3	70.8
Mkate (Bread)*	63.2	57.9	52.8	75.0	14.5	50.2
Ungalishe (composite flour)*	45.6	15.8	30.6	37.5	30.9	33.1
Ugali (stiff porridge)*	5.9	13.2	16.7	12.5	30.9	16.0
Visheti (puffed rice)	8.8	2.6	5.6	4.2	0.0	4.7
Togwa (non-alcohol drink)*	19.1	34.2	0.0	0.0	0.0	10.1
Pombe (alcohol drink)*	20.6	52.6	5.6	0.0	10.9	17.1
Pepeta knowledge as affected by						
Gender*			n = 57	n = 17	n = 13	n = 87
Male			35.3	68.4	69.2	53.0
Female			64.7	31.6	30.8	47.0
Age*^$^						
< 30 years (youth)			15.7	5.3	7.7	12.1
30–44 years (adult)			29.4	10.5	38.5	26.5
> 44 years (elder)			54.9	84.2	53.8	61.4

^a^ More than one answer possible

^#^no statistical analysis computed

*Significance difference (p< 0.05) among districts

^$^Significance difference (p < 0.05) in the overall respondents.

Source: Survey data, September 2017 –February 2018.

### 3.3 Processing season and availability

*Pepeta* is processed in large amounts twice a year, following the rice production calendar, which controls the availability of immature rice grains. The main production season is from April to July, i.e., the major rainy season when immature rice is sourced from both rainfed and irrigated fields, and the second season is from October to December when irrigated fields are the only source of rice. *Pepeta* processing as a source of household income was only mentioned by 50.0% (Kilombero) and 52.6% (Ulanga) of the respondents, the informants in the survey (Table 2), and was exclusively an activity of women in the visited areas. These findings are in line with previous research [33, 34], which underpins the important role women play in preserving and transferring traditional food processing knowledge from generation to generation in sub-Saharan region.

### 3.4 *Pepeta* processing

**Fig 3:**
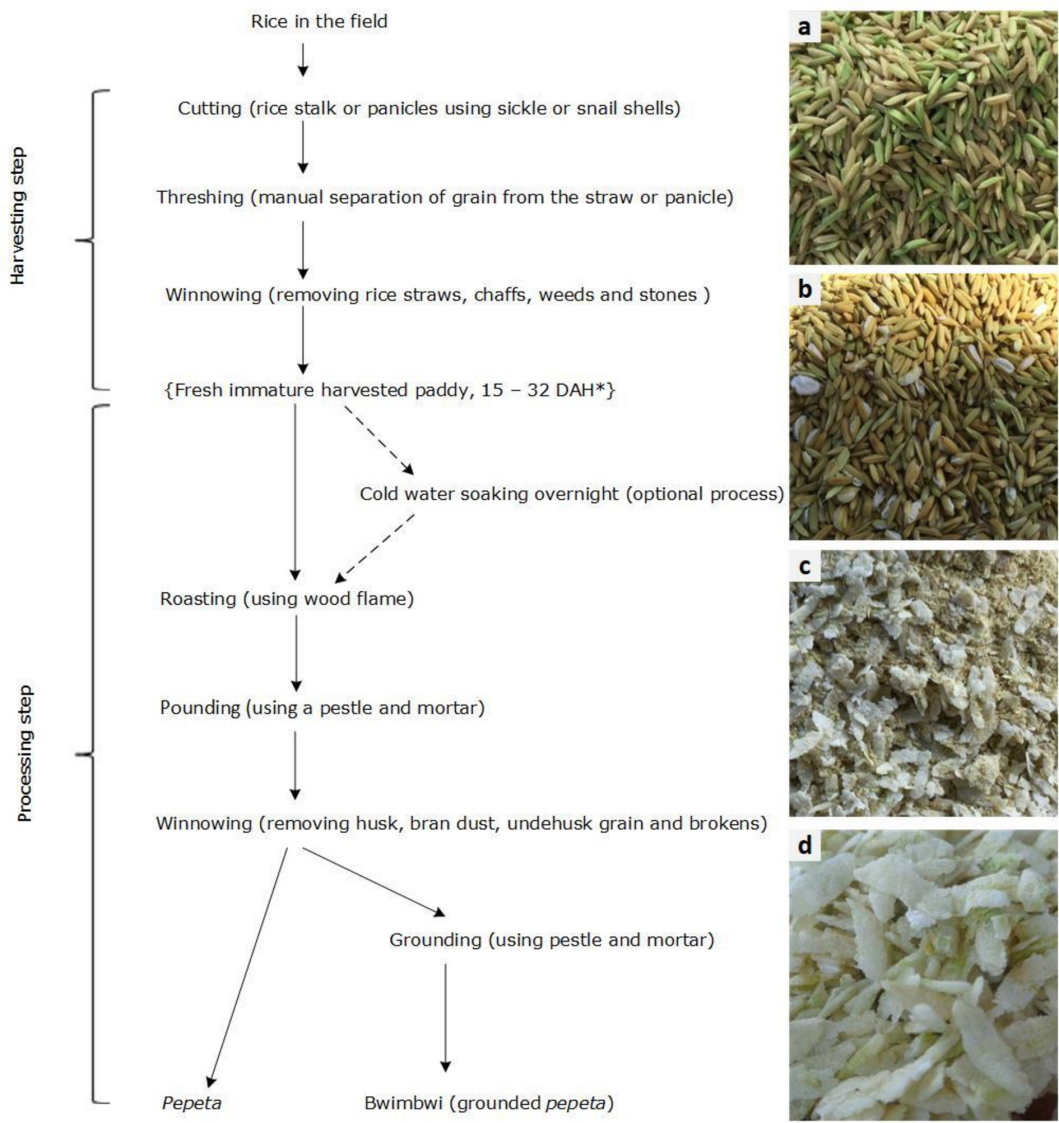
*Pepeta* processing flow diagram. DAH–days after 50% heading, **a**–fresh immature harvested paddy grains, **b**–roasted paddy grains, **c**–pounded paddy grains, and **d–**cleaned *pepeta* product.

*Pepeta* resembles flaked rice, but the use of freshly harvested immature rice grains make the product and production process distinct from many traditional and commercial flaked rice products in Asian and other rice–consuming countries [35]. The main processes involved in the production of *pepeta* are harvesting of immature rice grains, cold soaking, roasting, pounding, cleaning and packing (Fig 3). Though the production is mainly at household level, variations exist for *pepeta* production destined for sale compared to that intended for home consumption, mainly regarding processing quantity. For commercial production, 60–180 kg of *pepeta* per interviewed processor and less than 15 kg of *pepeta* per household for home consumption is processed during each cropping season. During *pepeta* processing, rice grains undergo several physical changes, including drying, a colour change from greenish yellow to bright yellow, dehusking and flattening (Fig 3A–3D). Table 5 shows the moisture content, duration, weight and temperature for various processing steps of *pepeta*.

**Table 5:** Moisture content, duration, weight and temperature at various processing steps of *pepeta* product.

Process step	Sample	Duration	Weight (kg)	Moisture content (%)	Temperature (^0^C)
Harvesting	Immature paddy (15–32 DAH)	2–12 h	18–36 (1–2 buckets)	31–36	Ambient temperature (21–31*)
Soaking	Immature paddy	Overnight (12–16 h)	18–36 (1–2 buckets)	--	Ambient temperature
Roasting	Immature paddy	3–8 min	0.25–0.41	9–15	Frame temperature (181–270)
					Vessel temperature (117–180)
					Paddy temperature (80–129)
Pounding	Roasted paddy	1–3 min	0.17–0.34	9–15	Ambient temperature
Cleaning	Pounded paddy		0.16–0.31		Ambient temperature
Packing/ storage	*Pepeta*		0.1–0.2	9–15	Ambient temperature

*Mkoma and Mjemah [36]. Source: Survey data, September 2017 –February 2018.

**Fig 4:**
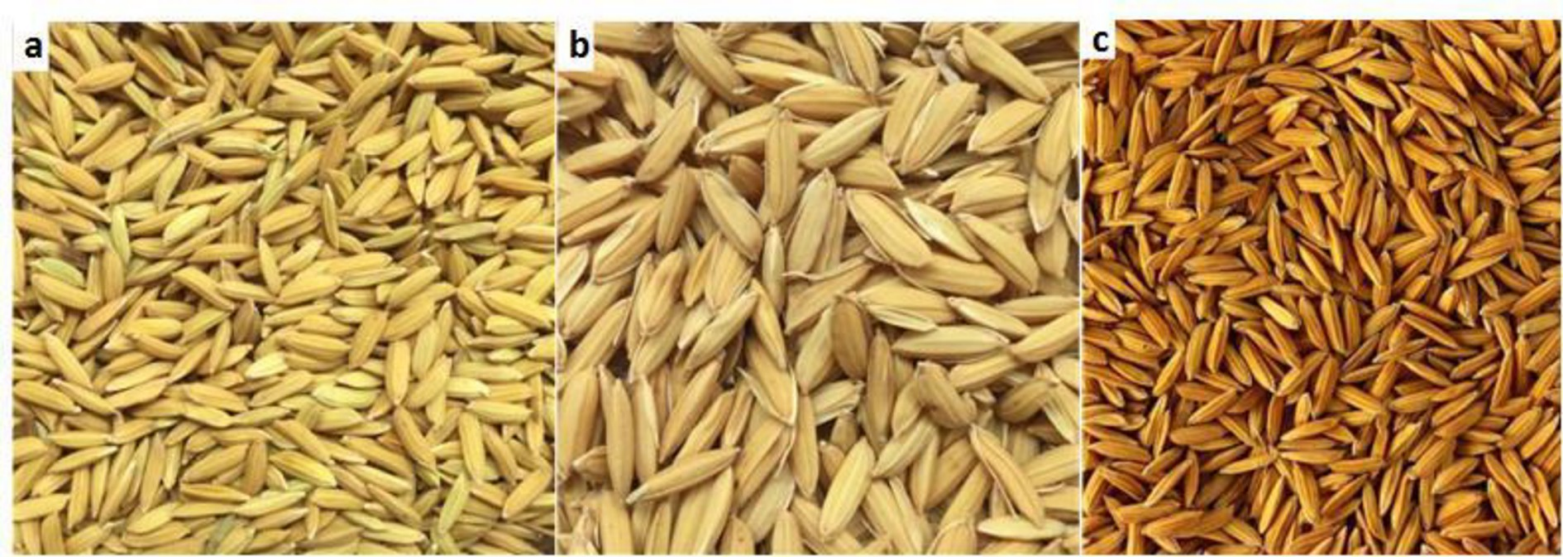
Most preferred rice verities used to process *pepeta* mentioned by respondents in the study area. a) TXD306/SARO 5, b) Mbawambili, and c) Kalamata. Source: Survey data, September 2017 –February 2018.

Selection of rice cultivar and optimum harvest time are critical steps for the quality and flavour characteristics of *pepeta*. Generally, *pepeta* is prepared from different aromatic landraces of rice (Table 6). Each informant cited at least four cultivars known to them and/or found in their locality. The most frequently cited cultivar was TXD306 mentioned by 100% and 95% of respondents in Kilombero and Ulanga districts, respectively. Participants were also asked to specify the most preferred cultivar. TXD306 (87.5% of respondents), Kalimata (7.1%) and Mbawambili (5.4%) were most preferred (Fig 4), and differ significantly (*x*^2^ = 7.554, df = 2, p = 0.023) between Kilombero and Ulanga respondents. Respondents considered TXD306, which is a semi-aromatic hybrid variety, to have quality traits and availability advantages over highly-aromatic landraces with a long growth cycle (i.e., Kalimata and Mbawambili). TXD306 can be cultivated twice annually, guarantees the availability of immature rice grains and hence *pepeta* processing. In addition, the awns of the Mbawambili variety tend to burn during roasting, introducing black spots and thereby affecting the general appearance of the *pepeta* product.

**Table 6:** Inventory of rice varieties commonly used for *pepeta* production in the surveyed area.

Local name (Swahili)	Category	Aroma	Citation in location (%)		Overall % (n = 55)
			Kilombero (n = 35)	Ulanga (n = 20)	
SARO5/TXD306	Hybrid	Semi-aromatic	100.0	95.0	98.2
Kalimata*	Landrace	Aromatic	0.0	90.0	32.7
Mbawambili*	Landrace	Aromatic	74.3	45.0	63.6
Nondo*	Landrace	Aromatic	45.7	0.0	29.1
Ngome*	Landrace	Aromatic	0.0	45.0	16.4
Wahipesa*	Hybrid	Non-aromatic	11.4	40.0	21.8
Supa-India	Landrace	Aromatic	25.7	35.0	29.1
Lawama*	Hybrid	Non-aromatic	34.3	10.0	25.5
Zambia	Landrace	Aromatic	28.6	25.0	27.3
Komboka*	Hybrid	Non-aromatic	22.9	0.0	14.5
Nyengo*	Landrace	Aromatic	17.1	0.0	10.9
Afa-Mwanza	Landrace	Aromatic	8.6	0.0	5.5
Kalimawangu	Landrace	Aromatic	5.7	0.0	3.6
Kisegese	Landrace	Aromatic	2.9	5.0	3.6
Most preferred variety*					
TXD306			94.3	75.0	87.5
Kalimata			0.0	20.0	7.1
Mbawambili			5.7	5.0	5.4

* Significance difference between Kilombero and Ulanga districts (p < 0.05).

Source: Survey data, September 2017 –February 2018.

**Fig 5:**
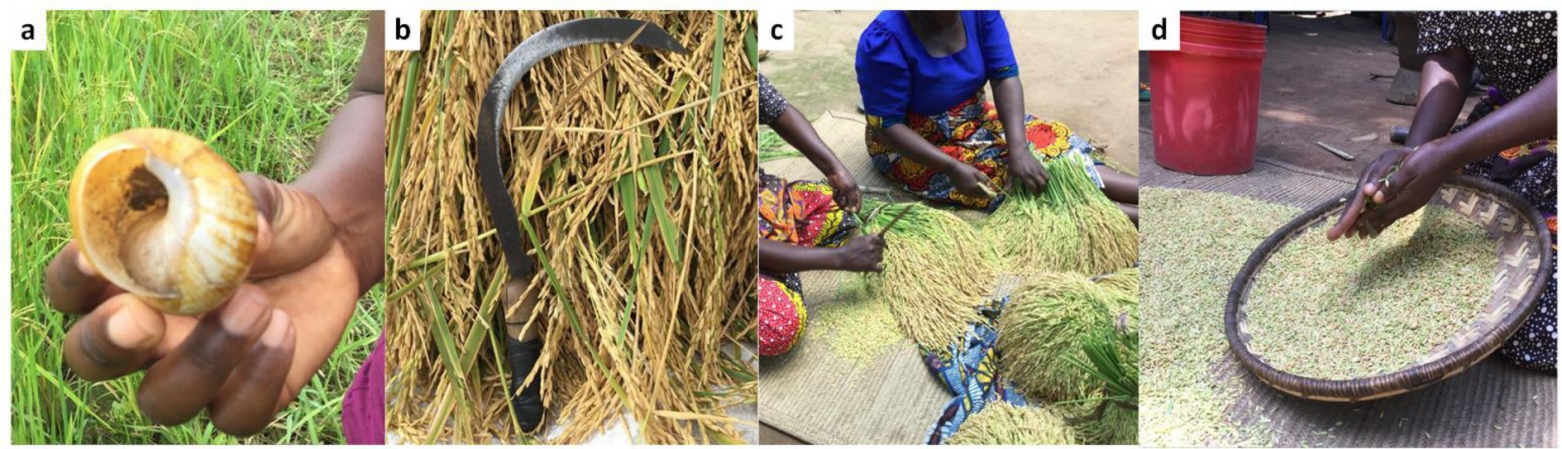
Harvesting of immature paddy rice grains during pepeta production using traditional equipment and methods. a) snail shell, b) sickle placed on harvest rice stalks, c) traditional hand threshing of harvested rice panicles, and d) traditional cleaning (winnowing) of threshed paddy. Survey data, September 2017 –February 2018.

*Harvesting of immature rice grains*. Harvesting immature rice destined for *pepeta* processing involves cutting, threshing and cleaning. Rice in the field is harvested by cutting rice panicles (60%) using a snail shell or cutting rice stalks (40%) using a sickle (Table 7, Fig 5A and 5B), and differed significantly (*x*^2^ = 16.042, df = 1, p = 0.000) between respondents of Kilombero and Ulanga districts. Harvesting by cutting rice panicles involves sorting of appropriate rice grains, leaving rice in the field that is considered unfit (too immature or too mature) for *pepeta* processing by processors. In contrast, the entire field is harvested when using sickles. Changes of leaf and panicle colour (92.7% of respondents) as rice matures, and biting through grain (25.5%) are common maturity indicators for optimum maturity of rice in the community, harvested two weeks (50.9% of respondents) or three weeks (32.7%) after 50% flowering of rice in the field. The colour of the grain seed coat changes from green to a distinct colour in accordance with rice cultivar at the onset of ripening, and grain endosperm hardens as it develops [37, 38]. Though no significant different in the indicators for optimum rice maturity, the suitability of rice in the field after optimum maturity varied significantly (*x*^2^ = 17.595, df = 2, p = 0.000) between respondents of Kilombero and Ulanga districts. According to respondents, rice in the field remained suitable for one (63.6% of respondents) to two weeks (34.5%) after attaining the optimum maturity level for *pepeta* processing.

**Table 7:** Overview harvesting of immature rice grains and its preparation before *pepeta* processing.

	Citation in location (%)		Overall % (n = 55)
	Kilombero (n = 35)	Ulanga (n = 20)	
Method of rice harvest*			
Manual by cutting rice panicles	80.0	25.0	60.0
Manual by cutting rice stalks	20.0	75.0	40.0
Indicators for rice maturity ^a^			
Changes of leaf and panicle colour	91.4	95.0	92.7
Grain biting	20.0	35.0	25.5
Days after seeding/transplanting	25.7	10.0	20.0
Days after 50% heading	25.7	25.0	25.5
Optimum harvest for pepeta			
Don’t know	14.3	20.0	16.4
2-weeks after 50% flowering	57.1	40.0	50.9
3-weeks after 50% flowering	28.6	40.0	32.7
Rice suitability after optimum maturity*			
1-week	82.9	30.0	63.6
2-weeks	14.3	70.0	34.5
3-weeks	2.9	0.0	1.8
Materials removed on harvested grains ^a^			
Unfilled kernels	88.6	100.0	92.7
Rice straws and chaffs	82.9	80.0	81.8
Stones/sands	37.1	35.0	36.4
Weed seeds*	42.9	0.0	27.3
Pre-treatment before roasting			
Paddy soaking overnight	68.6	65.0	67.3
Roasting of freshy harvested paddy	31.4	35.0	32.7

^a^ More than one answer possible

*Significance difference between Kilombero and Ulanga districts (p < 0.05). Source: Survey data, September 2017 –February 2018.

Threshing, the process that involves separating rice kernels from panicles but not removing the husk, is done immediately after cutting by hand (Fig 4C) or by beating with a stick on the paddy to maintain freshness of the harvested rice kernels. After threshing, rice grains are cleaned by a combination of winnowing and hand sorting (Fig 4D). Unfilled kernels (92.7% of informants), rice straws and chaffs (81.8%), stones (36.4%) and weed seeds (27.3%) were major unwanted materials removed during cleaning (Table 7). The unwanted materials removed during cleaning did not differ significantly between Kilombero and Ulanga respondents, except for weed seeds (*x*^2^ = 11.786, df = 1, p = 0.001). The moisture content of fresh harvested rice kernels ranged from 30–36% (Table 5).

*Soaking*. We observed cold water paddy soaking as an optional process, mainly in commercial production. It is done when harvesting until late in the evening to prolong freshness of harvested paddy until the next day. Table 7 indicates 67.3% of the respondents soaked freshly harvested immature paddy overnight, typically into 10–20 litre plastic containers. Paddy soaking is also said to soften and standardize the moisture content of harvested paddy kernels. Indeed, rice in the field matures heterogeneously, and large variations up to 46% in individual kernel moisture content at harvest have been reported [39, 40]. Generally, freshly harvested immature paddy is preferred, as it gives a more white-greenish colour. The soaked paddy is water drained at ambient temperature for about 2–4 hours before roasting.

**Fig 6:**
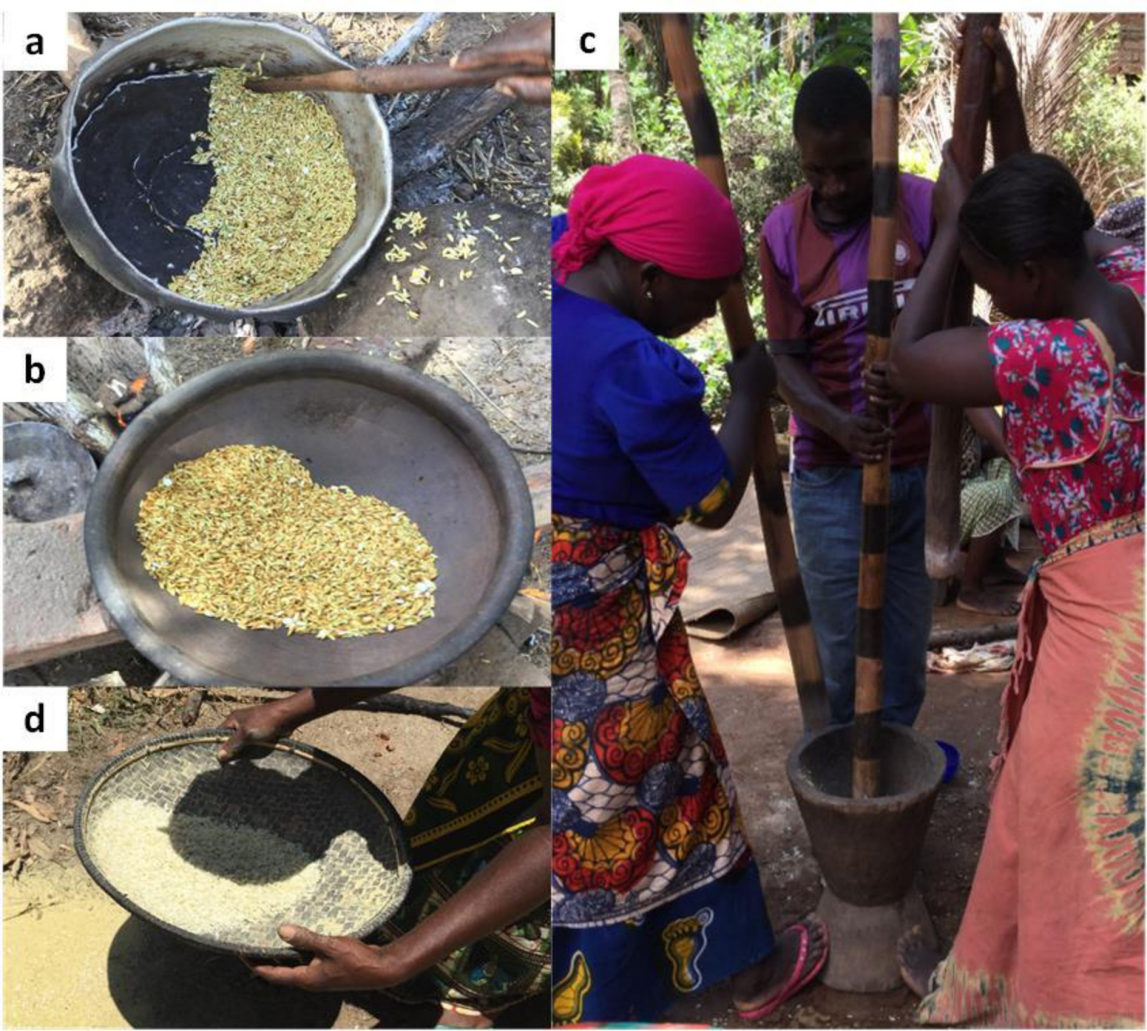
Major process steps of *pepeta* processing. a) hand roasting of paddy using aluminium pot, b) hand roasting of paddy using earthenware pot, c) traditional pounding using pestle and mortar, d) traditional cleaning. Source: Survey data, September 2017 –February 2018.

*Roasting*. In the study areas, fresh or soaked paddy is roasted in dry aluminium (Fig 6A) or earthenware (Fig 6B) pots, with continuous stirring using a big wooden spoon. We noticed no roasting medium such as sand or fine silt [35] used in many related flaked rice products. About 0.25 to 0.41 kg of paddy was roasted at a time for about 3–8 min. The roasting vessel was warmed up to 117–180°C and paddy reached a temperature from 80–129°C (Table 5). Table 8 show many of the respondents mentioned 1–5 min (49.1%) and 6–10 min (29.1%) as roasting duration. However, 56.4% of the informants indicated variations in the duration of roasting, depending on the amount of paddy roasted at a time, hotness of the firewood flame and efficiency of stirring.

**Table 8:** Overview of main pepeta processing steps in the study area.

	Citation in location (%)		Overall % (n = 55)
	Kilombero (n = 35)	Ulanga (n = 20)	
Roasting duration			
Don’t know	5.7	25.0	12.7
1 to 5 minutes	45.7	55.0	49.1
6 to 10 minutes	37.1	15.0	29.1
11 to 15 minutes	11.4	5.0	9.1
Variation in roasting duration	62.9	45.0	56.4
Indicators for terminating roasting ^a^			
Dryness of the grains*	97.1	80.0	90.9
Puffing of grains	62.9	45.0	56.4
Colour change of grains	60.0	40.0	52.7
Pounding duration			
Don’t know	8.6	25.0	14.5
1 to 5 minutes	62.9	60.0	61.8
6 to 10 minutes	28.6	10.0	21.8
11 to 15 minutes	0.0	5.0	1.8
Variation in pounding duration	68.6	60.0	65.5
Indicators for terminating pounding ^a^			
Absence of undehusked paddy grains	91.4	95.0	92.7
Flatness of the pounded grains	68.6	75.0	70.9
Number of pounding*	25.7	0.0	16.4
By-products of the pounding process ^a^			
Broken grains	74.3	75.0	74.5
Husk/brans	82.9	95.0	87.3
Undehusked grains	80.0	60.0	72.7
Packaging materials used ^a^			
Polyethylene bags	66.7	55.6	62.7
Plastic containers*	21.2	50.0	31.4
Paper bags*	24.2	0.0	15.7
Aluminium/earthenware pots	6.1	5.6	5.9
Most tedious processing step ^a^			
Harvesting (cutting)	25.7	5.0	18.2
Threshing	20.0	30.0	23.6
Roasting	11.4	10.0	10.9
Pounding	74.3	70.0	72.7

^a^ More than one answer possible

*Significance difference between Kilombero and Ulanga districts (p < 0.05). Source: Survey data, September 2017 –February 2018.

*Pepeta* processors depend on knowledge, experience and observations to determine when to terminate the roasting process. Dryness of the grains (90.9% of respondents), puffing of grains (56.4%) and colour changes of grains (52.7%) were common indicators to determine the end of the roasting process (Table 8, Fig 3B). There was no significance difference on various roasting parameters between Kilombero and Ulanga respondents, except for dryness of the grains (*x*^2^ = 4.526, df = 1, p = 0.033) a factor used as indicator for termination of roasting process. According to processors, dryness of the grain could be assessed by the ease of stirring due to a decrease in adhesion as grain dried during roasting, and/or easiness of dehulling when roasted grain is rubbed between hands. These findings differ from [35], who reported the initiation of a popping sound (puffing of grains) as the main indicator used for the termination of roasting in preparation of rice flakes.

*Pounding*. Pounding is the labour intensive and critical operation of *pepeta* processing, traditionally done by hand pounding the roasted paddy using a pestle and mortar (Fig 5C). Through pounding, the roasted paddy gets dehusked and flattened concurrently. Hand pounding is common practice when preparing traditional flaked rice from mature rice at household level in India [35]. Many respondents (65.5%) mentioned variations in the duration of pounding, where 61.8% and 21.8% reported a pounding duration of 1–5 min and 6–10 min, respectively (Table 8). However, during various *pepeta* processing demonstrations, about 0.17–0.34 kg of roasted paddy was pounded for 1–3 min by two or three people at a time (Table 5). We observed that pounding speed and hotness of the roasted paddy were important parameters. For effective processing, the pounding should immediately start and end while the roasted paddies are still hot. Even a slight delay from pounding may affect the thickness of the flattened grains and general appearance of the end product. Absence of undehusked paddy grains (92.7%) and flatness of the pounded grains (70.9%) were considered by respondents as major indicators for termination of the pounding process (Table 8, Fig 3C). No significant difference observed between Kilombero and Ulanga respondents in pounding parameters, except for number of pounding (*x*^2^ = 6.149, df = 1, p = 0.013) used as a factor to end the pounding process, mentioned by respondents (25.7%) in Kilombero district only.

*Cleaning and storage*. Fig 5D shows the cleaning process using the traditional winnowing method. The pounded grains are cleaned to remove by-products of the pounding process, namely broken grains (74.5% of respondents), husk/brans (87.3%), and undehusked grains (72.7%) (Table 8). In this study we found that the cleaning was done in two stages: first cleaning immediately after each pounding to remove brans, and a second cleaning at the end of *pepeta* production to remove remaining brans, broken and undehusked grains (Fig 3D). According to processors, any delay to remove brans immediately after pounding, affect whiteness and hence general appearance of the *pepeta* product. After cleaning, *pepeta* is stored in polyethylene bags (62.7% of respondents) and plastic containers (31.4%) of different sizes. Aluminium pots, earthenware pots and paper bags are seldom used. Although there was no significant difference in cleaning parameters, the packaging material used i.e. plastic containers (*x*^2^ = 4.483, df = 1, p = 0.034) and paper bags (*x*^2^ = 5.175, df = 1, p = 0.023) differed significantly between Kilombero and Ulanga respondents, with Kilombero being more sustainable-oriented. Paper bags which were only mentioned by respondents in Kilombero (24.2%), are considered as eco-friendly sustainable packaging material due to its biodegradability properties i.e. decompose by biological activity such as through bacteria or fungi into natural metabolic by-products [41].

### 3.5 Process efficiency and losses

**Fig 7:**
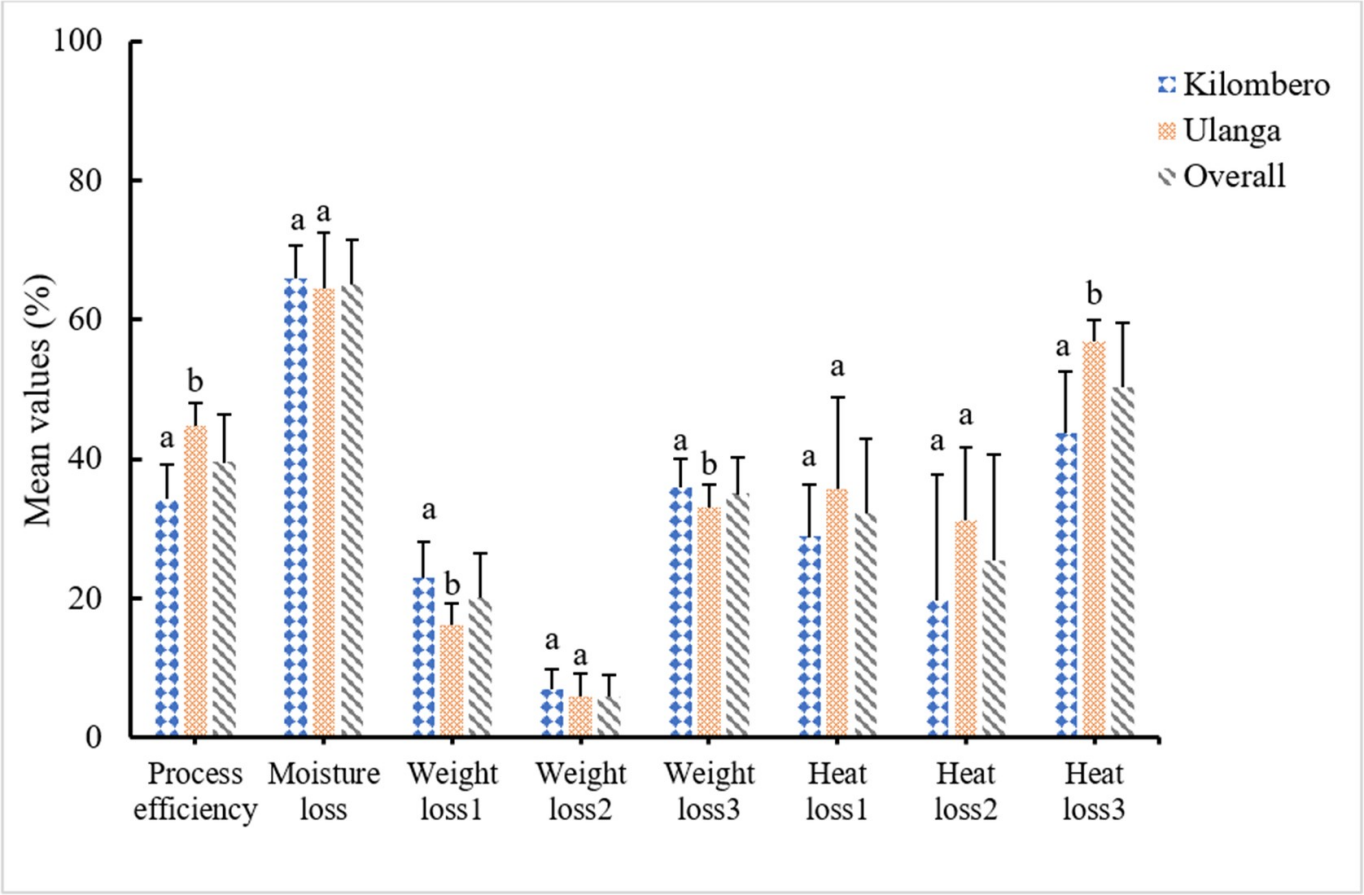
Mean values for process efficiency, and moisture, weight and heat losses which occur at various steps of *pepeta* processing; weight loss1 –loss at roasting, weight loss2 –loss at pounding, weight loss3 –loss at cleaning, Heat loss 1 –frame to vessel loss, Heat loss 2 –vessel to paddy loss, Heat loss 3 –Frame to paddy loss. Error bar in the chart represent standard deviation of mean value percentages. Bar with different letter are significant different at p < 0.05 for each parameter. Source: Survey data, September 2017 –February 2018.

Several terms are used by experts to evaluate the efficiency of processing methods. In this study we use *pepeta* recovery percentage to evaluate the process efficiency of *pepeta* processing. Based on the rice milling recovery concept [42], we define *pepeta* recovery (i.e. process efficiency) as the percentage of *pepeta* yield based on the initial paddy weight before roasting. The survey found 40% recovery when immature paddy was processed into *pepeta* (Fig 7), a lower value than the 50–60% [43] when mature dried paddy is milled. The apparent low yield of *pepeta* attributed to 35% weight loss due to remove of unwanted materials (husk, bran dust, broken and undehusked paddy grains) during cleaning, 20% weight loss during roasting, and 6% weight loss due to grain scattering during pounding. High moisture loss (65%) was the main factor contributing to weight loss during roasting. However, processing efficiency, loss at roasting and cleaning as well as heat loss from flame to paddy differed significantly between Kilombero and Ulanga respondents, with Ulanga being more efficiency compared to Kilombero.

### 3.6 *Pepeta* trade

In the surveyed area, *pepeta* trade is largely carried out in the Morogoro region, with by far the largest proportion of the commercial production and distribution chain dominated by women. The trade starts in Kilombero and Ulanga districts, which are the centres for *pepeta* production in the region. In these districts, processors bought immature paddy directly from the field, which was sold at a price almost three times that of dried mature paddy. This could be a way to offset losses due to the fact that when harvesting immature rice the yield is lower than for mature rice [44]. According to respondents, prices varied from one place to another, and depending on seasonal availability: lower during bumper harvest and higher during scarce supply. *Pepeta* was sold mainly along roadside selling centres (100% of respondents), seldomly at local markets (18.2%) and train stations (10.9%) (Table 9). Although there was no significant difference in roadside selling centres, *pepeta* selling at train stations differed significantly (*x*^2^ = 3.848, df = 1, p = 0.049) between Kilombero (17.1%) and Ulanga (0.0%) respondents. It is important to note that Ulanga district is not accessible by train as Kilombero district. The study found three major *pepeta* roadside selling centres, namely *Ruaha getini* (07.66^o^S 036.97^o^E) and *Mang’ula kona* (07.85^o^S 036.89^o^E) in Kilombero district, and *Kivukoni getini* (08.20^o^S 036.69^o^E) in Ulanga district. The processors who are also traders, have formed their groups, which regulate the number of processors selling the product in a daily rotation routine. This is because there are more processors than the capacity of the vending centre. However, processors complained about a lack of reliable markets opportunities, indicating the challenge of *pepeta* distribution chain and the potential to improve the trading network.

**Table 9:** Overview of *pepeta* trade in the study area.

	Citation in location (%)		Overall % (n = 55)
	Kilombero (n = 35)	Ulanga (n = 20)	
*Pepeta* uses			
As snack	97.1	100.0	98.2
As breakfast	2.9	0.0	1.8
Reason for processing *pepeta*			
Vending only	54.3	45.0	50.9
Vending and household consumption	45.7	55.0	49.1
*Pepeta* selling location ^a^			
Roadside^#^	100.0	100.0	100.0
Local market	22.9	10.0	18.2
Train station*	17.1	0.0	10.9
Middle persons/mobile call out	5.7	0.0	3.6

^a^ More than one answer possible

^#^No statistical analysis computed

*Significance difference between Kilombero and Ulanga districts (p < 0.05). Source: Survey data, September 2017 –February 2018.

The price of *pepeta* did not change during the processing season and was twice that of milled white rice. This is illustrative of the importance of adding value to agricultural produce for household income generation and hence livelihood and food security improvement [2, 45] This study found that *pepeta* was also sold to the neighbouring municipalities such us Morogoro and Dar es Salaam by middle-persons (3.6% of respondents). Processors mentioned to produce *pepeta* for either selling (50.9%) or both selling and household consumption (49.1%). Though the contribution to household income has not yet been documented, most processors cited that it covers by far the greatest proportion of their daily needs such as food, clothes and school fees.

### 3.7 Product quality criteria and acceptability

**Fig 8:**
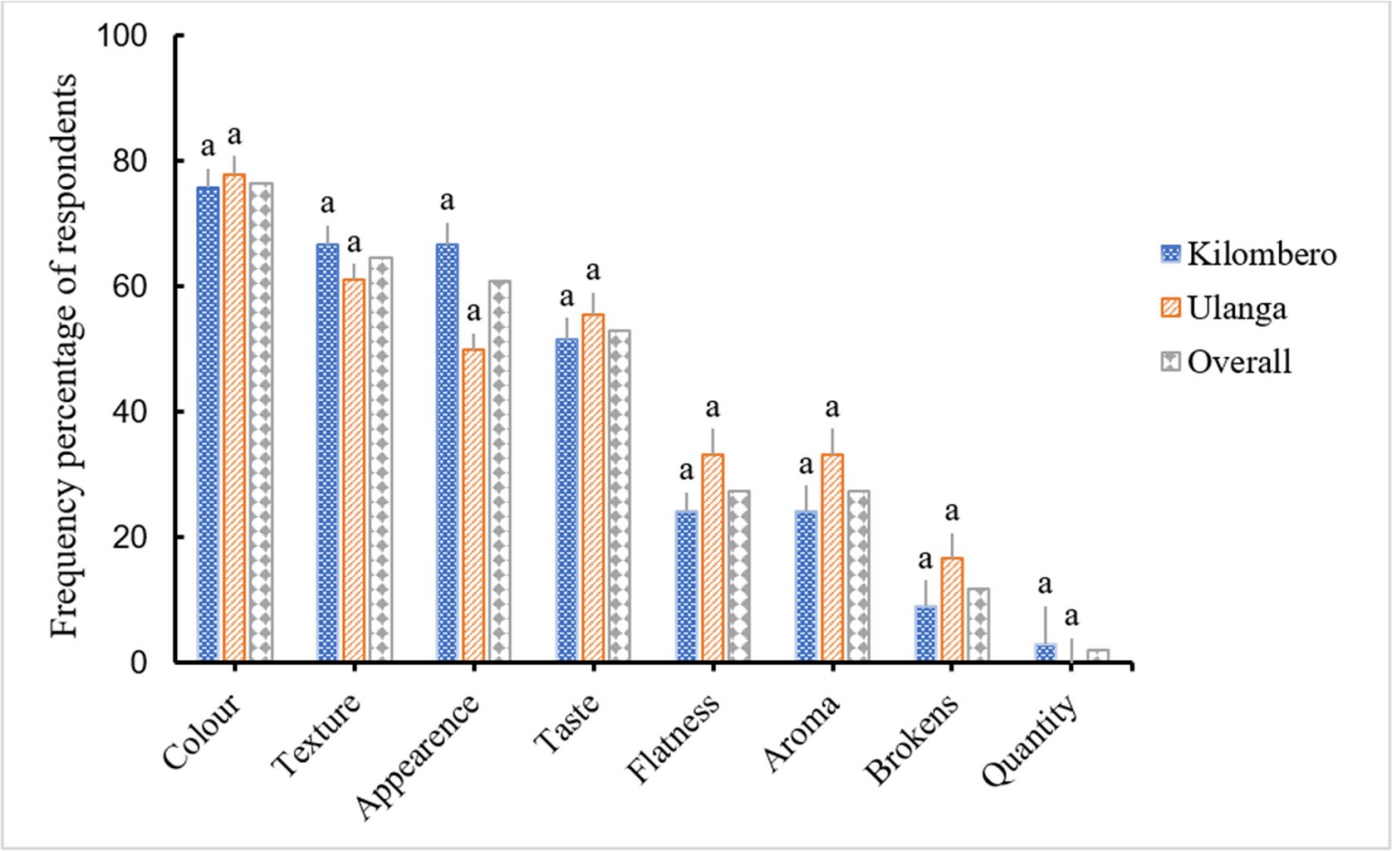
Factors considered by consumers when buying *pepeta* product in the study area. Kilombero–Kilombero district, Ulanga–Ulanga district. Bar with different letter are significant different at p < 0.05 for each parameter. Source: Survey data, September 2017 –February 2018.

Fig 8 indicates factors considered by respondents in the study area when buying and consuming *pepeta*. According to respondents, major factors considered were colour (76.5% of respondents), general appearance (60.8%), texture (64.7%) and taste (52.9%). In addition, aroma, flatness and number of broken grains were also considered. Location had no effect on quality criteria considered by respondents between Kilombero and Ulanga district. Colour was mentioned as a highly critical factor in the focus group discussions as well. According to the interviewees, a white-greenish colour (Fig 3D) is most preferred as it indicates that the product is made from immature rice grains. The informants cited general appearance to include presence or absence of black spots, husks, bran, undehusked grain, dust, broken, and flatness of pounded grains. We observed that the general appearance and colour of *pepeta* product act as eye-catching traits, highly differentiate *pepeta* products among processors and play a key role in consumer decisions to taste the product before buying. This is possible because *pepeta* is marketed in unpacked form, whereby consumers are invited to taste the product before buying.

After tasting, flavour and texture played decisive roles in consumers’ buying decisions. According to interviewees, a good *pepeta* texture is not too crunchy, moderately soft but not too sticky and thin. Thick and hard *pepeta* was reported to cause jaw aching even when consumed only for a short time. Generally, the use of immature rice grains gives a butter-like popcorn flavour to *pepeta*, which is highly appreciated by many consumers (98%) in the surveyed areas. Indeed, the maturity level of cereal grain has been associated to the taste of *Firiks* (or *Frekeh*), a traditional product prepared from immature wheat [16, 46]. We found that the most common way of consuming *pepeta* is as a snack (98.2% of respondents), whereas 1.8% used *pepeta* as breakfast. In this study, snack refers to consumption as a leisure activity rather than as a meal, with no specific time.

### 3.8 Pepeta production problems

**Fig 9:**
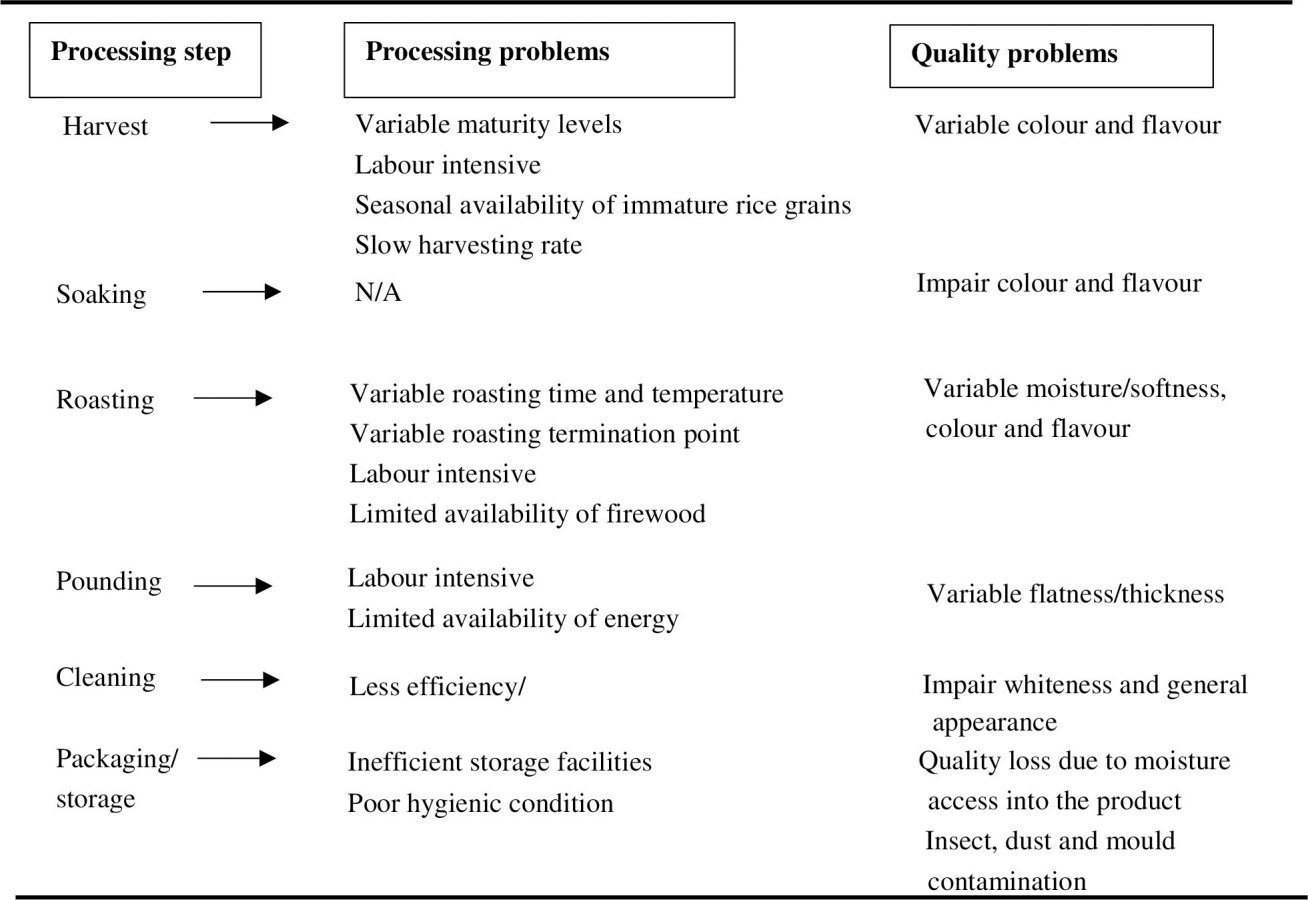
*Pepeta* processing and quality problems identified by respondent in study area. Survey data, September 2017 –February 2018.

Fig 9 presents a general overview of *pepeta* processing and quality problems identified by respondents in the study area. *Pepeta* processors used colour changes of rice grains and hardening of endosperm as criteria to identify the optimum rice maturity level for *pepeta* processing. This method is very subjective, resulting in variations in maturity levels of the harvested rice grains, and hence colour and flavour of *pepeta* end products among processors.

Harvesting by cutting and threshing immature rice grains is done under wet conditions, a period when water in the rice fields is still needed for crop growth. Generally, it is difficult to control the water level within individual rice fields due to poor infrastructure. This makes the use of local harvesting tools and methods inevitable as mechanised tools cannot be used in wet conditions, making the process laborious and time-consuming. In some occasions, processors soak immature paddy in cold water to extend the freshness of harvested paddy until the next day when harvesting ends late in the evening. However, a focus group interviewee said that soaking imparted undesirable colour changes and off-flavour to the end product, similar complaints as reported by [35].

Dryness, puffing and colour changes of rice grains are important factors used by processors to determine the end of the roasting process, a subjective method based on past experience and visual observations. In addition, these factors highly depend on the amount of paddy being roasted, temperature and stirring speed. Though the amount of paddy is easily controlled by roasting a known amount, maintaining a constant temperature and stirring speed is difficult due to the use of firewood and manual stirring. Properties such as wood density, moisture content, flammability, flame brightness, and flaming period affect flame temperature [47]. These are not considered much by processors as the type of firewood used in the study area is based on availability.

Usually, roasting is done in open air, a situation which further aggravates the problem of uncontrolled heat distribution during roasting due to air velocity. An uncontrolled heat flow and inefficient stirring result in over-roasted paddy due to high heat or under-roasted paddy due to false puffing of rice grain, respectively. This causes a poor quality of the end product, with defects concerning moisture content, softness, colour and flavour (Fig 7). To prevent such issues, processors have to continuously stir while regulating the amount of chopped wood to maintain a proper heat flow during roasting, a tiresome process according to processors.

About 72.7% of the respondents mentioned pounding as the most tedious process step, at least two times more frequent than the remaining processing steps: threshing (23.6%), cutting (18.2%) and roasting (10.9%) (Table 8). Application of labour serving technology like roaster and rice flaking machine [48] will substantially relief the workload. To achieve the desired flatness, processors have to manually pound roasted paddy at a high speed to finish the process quickly while the paddies are still hot. This is because cold pounded rice grains do not flatten; instead normal milled white rice kernels are obtained. Processors complained about physical discomfort, including hand, back and chest pain.

Respondents mentioned an impairment of whiteness and general appearance of the end product when cleaning is delayed after pounding. Other constraints include quality degradation due to moisture uptake by the product, and contamination by insects, dust and mould as a result of poor storage facilities and hygienic conditions. *Pepeta* as a dried product, tends to absorb water from the surroundings. To maintain product quality, *pepeta* was stored in plastic containers with tight-closing lids and kept in cool and dry conditions. In addition, respondents in the focus group discussion mentioned to re-sundry *pepeta* to extend its shelf life when stored for a long period, especially during the rainy season. The results concur with [33], who reported re-sundrying of traditionally prepared dry monkey orange (*Strychnos* spp.) products to extend shelf life. Wet and high humidity storage conditions facilitate clump formation, mould growth, discolouration, a bad smell and bitter taste; factors that were much considered by consumers when rejecting a *pepeta* consignment.

### 3.9 General utilization of immature cereal-based products

Cereals are the major staple food for many people. Wheat, maize and rice comprise at least 75% of the world’s grain production [49]. Consumption of immature maize as a roasted product or boiled whole kernel, and/or traditional processed products is common in Africa [50, 51]. *Mohlefe* or *malitsibana* is a common green maize bread in Lesotho, prepared by wet-milling maize kernels to a thick dough, shaped into cob-like forms, covered with maize leaves and steamed until completely cooked [51]. Similar to *pepeta*, the preparation of *mohlefe* is done during the harvest season when households are waiting for crops to mature in the field. Degree of maturity and varietal differences significantly impact the quality properties of *mohlefe*. Preferred attributes are a whitish or yellowish colour, depending on the maize variety used, and an intense aroma of immature green maize kernels [51]. Contrary to *pepeta*, salt is added during preparation of *mohlefe*, and this food is mostly consumed as breakfast.

*Firik* (also known as *frikeh* or *frekeh* or *freekah*) is a common immature whole wheat-based food consumed in the Middle East and North African countries [16, 52]. Processing knowledge and quality attributes of *firik* have been extensively studied [16, 46, 53–55]. To prepare *firik*, immature wheat ears are scorched or roasted on open fire, sundried, threshed, after which the kernels are separated from the hulls and cracked [46]. Similar to *pepeta*, harvesting time, wheat cultivar and processing conditions determine the quality attributes of *firik*. The best harvest time for *firik* ranges from the late-milk to mid-dough stages, which gives a better taste than for the ones processed at the full ripe stage. Hard durum wheat (*Triticum durum*) is preferred for *firik* production. Scorching gives the *firik* its unique, appetizing smocked flavor [16]. Generally accepted high quality *firik* is plump, firm when fresh, slightly burnt, green when dried, containing few remains of the pleas, lemmas, glumes, and free from stones and debris [52]. *Firik* is widely used as an ingredient in the preparation of some specific meals, and especially consumed with meat, tomatoes, stuffed squash, eggplant, grape leaves, and chicken broth [16, 46].

Novel processing of immature grains to increase their applications in food industry has been documented. Previous study [56] assessed the application of fluidized-bed coating technology in improving the health benefits i.e. antioxidant activities of Khao Mao cereal product (a traditional puffed pounded-unripe rice in Thailand). Yilmaz *et al*.[18] investigated the potential use of infrared radiation to unfold the limitation use of immature rice grains due to high rancidity rate compared to mature rice grains. Infrared stabilized immature rice grain flour has been used in preparation of extruded rice products [57], gluten free bread [17], and Tarhana, a cereal based fermented food [58]. Çetin-Babaoğlu *et al*. [59] and Pepe *et al*. [60] evaluated the application of immature wheat flour in straight-dough and sour-dough (fermentation) processes for production of wheat bread. In addition, fermentation technology has been widely used to improve the nutritional quality of other immature cereal-based products like green spelt wheat tempe [61], and green maize (*melie*) and green sorghum (*senkhoana*) breads [51].

## 4. Conclusions and recommendations

This study presents the current use, potential and challenges of *pepeta* and its traditional processing knowledge on promoting physical and economic accessibility to early season food, which are main aspects of food security, at household level in Tanzania: “Food security is achieved when all people, at all times, have physical and economic access to sufficient, safe and nutritious food that meets their dietary needs and food preferences for an active and healthy life” [14]. Selling immature rice and *pepeta* provides economic benefits for local farmers and processors since these products are commonly sold at prices that are about three times higher than for mature paddy and white rice. The premium price earned contributes to the livelihood of the household.

However, the current traditional processing method has disadvantages: the process is slow, laborious and not suited for upscaling as this cannot guarantee a consistently good quality *pepeta* product. There are no standards for rice harvesting, and processing conditions and parameters. These problems affect its full utilization across sub-Saharan Africa, further aggravate food insecurity in the region. In order to develop a nutritious high sensorial quality *pepeta* product with better shelf life and ease of portability, research on the factors which influence its quality including nutritional, physico-chemical, rheological and aroma properties is of paramount importance. Research to optimise the major process parameters of *pepeta* processing, such as maturity level and moisture content of immature rice grains, and roasting conditions (i.e., temperature and time), for high nutritional and sensorial quality *pepeta* product should not be neglected by researchers. Ultimately, assessing proper preservation, packaging, storage conditions and shelf life stability, and possibility for value addition of *pepeta* product can create new markets in the cities and towns in the country and outside Tanzania. As these food technological problems and solutions are products specific, the social culture, food culture and socio-economic impact of *pepeta* improvement must be considered by the researchers.

## Supporting information

S1 Questionnaire Indigenous processing knowledge and consumers’ preferences on *pepeta*. (DOCX)

S1 Checklist Indigenous processing knowledge and consumers’ preferences on *pepeta*. (DOCX)
