# Supplementary material for: Utilization of Pepeta, a locally processed immature rice-based food product, to promote food security in Tanzania
Source: PLoS One. 2021 Mar 3;16(3):e0247870. doi: 10.1371/journal.pone.0247870 (PMC7928465; doi:10.1371/journal.pone.0247870)
Supplement: S1 Questionnaire — (DOCX) [file pone.0247870.s001.docx]

**S1 Questionnaire. Indigenous processing knowledge and consumers’ preferences on *pepeta***

**GPS coordinates:**

____________________

**Questionnaire number**

**|___|___|___|___|___|___|___|**

**| Dist | Ward | HH |**

“Hello. How are you? My name is...…………………………………….. and I am working with a team from................................... We are conducting a survey of households in this district, and your household was randomly chosen to be interviewed in this community. We would like to ask you some questions about Pepeta processing practices, selling and consumption. This information will be beneficial in improving processing, nutritional and sensory qualities and its availability all year round for potential improvement for food security. May I ask 60 minutes of your time to answer these questions? **The collected information will be used solely for research purposes.**

**Part I: Questionnaire for general Rice/Pepeta actors**

**Section 1: Identification and socio-demography characteristics**

| **N°** | **Questions** | **Answers** | **Code** |
| --- | --- | --- | --- |
|  | Name of interviewer |  |  |
|  | Date of interview |  |  |
|  | Time | Start time:  Finish time: |  |
|  | District |  |  |
|  | Ward |  |  |
|  | Village & Kitongoji |  |  |
|  | Main interview language | 1. English 2. Swahili 3. Other (specify) ………………………. |  |
|  | Name and surname (interviewee) | …………………………………………………………………….  ......................................................................................................... |  |
|  | Gender | 1. Male 2. Female |  |
|  | Marital status | 1. Single 2. Married 3. Divorced 4. Widow/ Widower |  |
|  | Age in years | ……………………………… |  |
|  | Education level | 1. No education 2. Primary school 3. Secondary “O” level 4. Secondary “A” level 5. Vocational training 6. Tertiary (diploma or degree) 7. Other (specify) …………………… |  |
|  | Main source of income  (*possible to give multiple responses in order of importance*) | 1. Rice cultivation only 2. Agricultural activities (crop cultivation and livestock keeping) 3. Pepeta processing 4. Casual labour 5. Remittances 6. Petty trade (other than Pepeta) 7. Other (specify) ……………. |  |
|  | Are you household head ? | 1. Yes 2. No |  |
|  | Position of the household | 1. Husband 2. Wife 3. Child 4. Other (specify) ……………………. |  |

**Section 2: Rice knowledge and activity**

| **N°** | **Questions** | **Answers** | **Code** |
| --- | --- | --- | --- |
|  | What crops do you cultivate  *(possible to give multiple responses)* | 1. Rice 2. Maize 3. Sugarcane 4. Sunflower 5. Banana 6. Cotton 7. Groundnut 8. Sweet potato 9. Cassava 10. Beans 11. Other (specify)…………………… |  |
|  | Which one is your main crop(s) for cultivation ?  *(possible to give multiple responses in order of importance)* | 1. Rice 2. Maize 3. Sugarcane 4. Sunflower 5. Banana 6. Cotton 7. Groundnut 8. Sweet potato 9. Cassava 10. Beans 11. Other (specify)…………………… |  |
|  | What are rice varieties that you are familiar with?  *(possible to give multiple responses in order of importance)* | 1. Supa India 2. Saro 5 (TXD 306) 3. Kalamata 4. Kisegese 5. Mbawambili 6. Nondo 7. Zambia 8. Komboka 9. Tai 10. Baraka 11. Other (specify)……………….. |  |
|  | What are common rice varieties in this locality?  *(possible to give multiple responses up to three main varieties in order of importance)* | 1. Supa India 2. Saro 5 (TXD 306) 3. Kalamata 4. Kisegese 5. Mbawambili 6. Nondo 7. Zambia 8. Komboka 9. Tai 10. Baraka 11. Other (specify)……………….. |  |
|  | Can you identify different rice-based food products that you are familiar with?  *(possible to give multiple responses)* | 1. Pepeta 2. Polished/white rice 3. Brown rice 4. Parboiled rice 5. Mchopeko (traditional parboiled rice) 6. Vitumbua (rice dough) 7. Mkate wa kumimina (rice bread) 8. Unga wa lishe (composite flour) 9. Visheti (biscuits) 10. Other (specify) …………….. |  |
|  | If Pepeta is among answers, which category do you fit in relation to Pepeta? (*For each category* ***>>*** *corresponding section*) | 1. Processor (Part II) 2. Seller (Part III) 3. Consumer ( Part IV) |  |

**Part II: Questionnaire for Pepeta processors**

**Section 3 :** **General information on Pepeta processing**

| **N°** | **Questions** | **Answers** | **Code** |
| --- | --- | --- | --- |
|  | Can any rice variety be used used to perepare Pepeta? | 1. Yes 2. No |  |
|  | What common rice varieties are used for Pepeta production and why? | ………………………………………………………………..  ………………………………………………………………..  ……………………………………………………………….. |  |
|  | What is the most used/preferred variety? Why? | ………………………………………………………………..  ………………………………………………………………..  ……………………………………………………………….. |  |
|  | What are the characteristics of the most used rice variety? (*description related to the appearance, colour, size, etc*) | 1. Appearance (size)   ……………………………………………………………  ……………………………………………………………  ……………………………………………………………   1. Color   …………………………………………………………………………………………………………………………   1. Aroma (aroma, semi aroma, none aroma)   …………………………………………………………………………………………………………………………   1. Other (specify)   ……………………………………………………………  …………………………………………………………… |  |
|  | What is the source of rice used for Pepeta processing? | 1. Own production 2. Other farmers/producers 3. Both |  |
|  | If not from your own production, how do you pay? | 1. In kind (free) 2. Barter trade 3. Cash |  |
|  | If cash, how much do you pay per given load? (in TZS per kg*)  **estimate the load in kg* | 1. During plenty supply period….………………… 2. During shortage supply period.…………………………. |  |
|  | Do you process the rice immediately after purchasing/harvest? | 1. Yes 2. No |  |
|  | If not, how long do you store them before processing? | …………………………………………………………………  ………………………………………………………………… |  |
|  | Describe the place that you store them (immature paddy) and the materials used? | …………………………………………………………………  …………………………………………………………………  …………………………………………………………………  ………………………………………………………………… |  |
|  | How many Pepeta processing season (using immature rice) per year? | 1. Once a year 2. Twice a year 3. Other (specify).......... |  |
|  | What is the product processing frequency per season? | 1. Once 2. Two times 3. Three times 4. Four times 5. Other (precise).......................... |  |
|  | How much of the Pepeta (kg) do you produce per season? | …………………………………… |  |

**Section 4: Unit operations on Pepeta processing**

| **N°** | **Questions** | **Answers** | **Code** |
| --- | --- | --- | --- |
|  | **Cutting/harvesting** |  |  |
| - - 1. 3.1 | How do you know when the crop is mature for harvest for Pepeta processing? (*possible to give multiple answers*) | 1. Number of days after seeding/transplanting (DAS/DAT) 2. Number of day after 50% heading/flowering (DAH) 3. Leaf and panicle colouration 4. Biting through grain 5. Moisture meter reading 6. Other (specify) ……………. |  |
|  | What is the maturity level (DAS/DAT/ DAH as estimated by the processor) of rice used for Pepeta production ? (*to be confirmed during practical demonstration)* | …………………………………………………………..  ………………………………………………………….. |  |
|  | How long (days or weeks) is paddy in the field still suitable for Pepeta processing from the first harvest/ maturity level ? | …………………………………… |  |
| - - 1. 3.3 | What is the method used to harvest? | 1. Manual by cutting rice stalks 2. Manual by cutting rice panicles 3. Mechanical 4. Other (specify)…………… |  |
|  | **Threshing** (*involves separating the grain from panicles but not removing the husk*) |  |  |
|  | Do you thresh the paddy immediately after cutting/harvest? | 1. Yes 2. No |  |
|  | If yes, why? | ………………………………………………………………  ………………………………………………………………  ……………………………………………………………... |  |
|  | If not, how long do you store them (harvested paddy) before threshing, and why? | ………………………………………………………………  ………………………………………………………………  …………………………………………………………….... |  |
|  | What is the method used to thresh? | 1. By hand 2. Stick directly on paddy 3. Paddy directly on piece of trunk 4. Stick on the bag of paddy 5. Mechanical 6. Other (specify) ……………. |  |
|  | What difficulties encountered during threshing? | ………………………………………………………………  ………………………………………………………………  ………………………………………………………………  ……………………………………………………………… |  |
|  | **Sorting** (*pre-cleaning of threshed paddy before roasting*) |  |  |
|  | Do you clean the paddy immediately after threshing? | 1. Yes 2. No |  |
|  | If yes, why? | ………………………………………………………………  ………………………………………………………………  ……………………………………………………………… |  |
|  | If not, how long do you store them (threshed paddy) before cleaning, and why? | ………………………………………………………………  ………………………………………………………………  ……………………………………………………………… |  |
|  | Describe the place that you store them and the materials used? | ………………………………………………………………  ………………………………………………………………  ……………………………………………………………… |  |
|  | What are the common unwanted materials removed from threshed paddy? (*possible to give multiple responses*) | 1. Unfilled grain 2. Sand/stones 3. Rice straws/leafs 4. Weed seeds 5. Rice grain of different variety 6. Over matured rice grain 7. Other (specify)………………… |  |
|  | What is the method used to clean? (*possible to give multiple responses*) | 1. By hand (physical sorting) 2. Winnowing (traditional cleaning using wind) 3. Mechanical 4. Other (specify) ……………. |  |
|  | What difficulties encountered during cleaning? | ………………………………………………………………  ………………………………………………………………  ……………………………………………………………… |  |
|  | **Roasting** (*heating of cleaned moist paddy*) |  |  |
|  | Do you roast the paddy immediately after cleaning? | 1. Yes 2. No |  |
|  | If yes, why? | ………………………………………………………………  ……………………………………………………………… |  |
|  | If not, how long do you store them (cleaned paddy) before roasting, and why? | ………………………………………………………………  ………………………………………………………………  ……………………………………………………………… |  |
|  | Describe the place that you store them and the materials used? | ………………………………………………………………  ………………………………………………………………  ……………………………………………………………… |  |
|  | What factors used to determine end of roasting process? (*possible to give multiple responses in order of importance*) | 1. Change of colour 2. Dryness of roasted rice grain (moisture content) 3. Roasting duration/time 4. When rice grains start puffing/popping 5. Other (specify)………………… |  |
|  | What is the roasting duration (time in minutes per specific amount roasted as estimated by the processor)? (*to be confirmed during practical demonstration)* | ………………………………………………………………  ……………………………………………………………… |  |
| - - 1. I | Does roasting duration vary with maturity level/ moisture content of raw immature paddy? | 1. Yes 2. No   If yes, explain (how) ……………………………………….  ……………………………………………………………..  ………………......................................................................  ……………………………………………………………… |  |
|  | What is the roasting method used? | 1. Oven air 2. Oven air + sand 3. Flame (wood, charcoal, electric plate, gas, etc ) 4. Flame + sand 5. Other (specify) ……………. |  |
|  | What is your source of energy for heating/roasting?  (*possible to give multiple responses*) | 1. Firewood 2. Livestock and crop residues (manure/rice husk) 3. Charcoal 4. Kerosene/paraffin 5. Electricity 6. Gas 7. Other (specify) ……………….. |  |
|  | How accessible is your energy source? | 1. Easy 2. Fairly easy 3. Difficult 4. Extremely difficult |  |
|  | How do you pay for energy source? | 1. No payment 2. Barter trade 3. Cash 4. Other (specify)……………….. |  |
|  | If cash, how much do you pay per given load of energy per month in TZS? (*for Pepeta processing only, if possible*) | …………………………………….. |  |
|  | What difficulties encountered during roasting? | ………………………………………………………………  ………………………………………………………………  ……………………………………………………………… |  |
|  | **Pounding** (*involve remove of husk and flattening of the grains at the same time*) |  |  |
|  | Do you pound the paddy immediately after roasting? | 1. Yes 2. No |  |
|  | If yes, why? | ………………………………………………………………  ………………………………………………………………  ……………………………………………………………… |  |
|  | If not, how long do you store them (roasted paddy) before pounding, and why? | ………………………………………………………………  ……………………………………………………………… |  |
|  | Describe the place that you store them and the materials used? | ………………………………………………………………  ………………………………………………………………  ………………………………………………………………  ……………………………………………………………… |  |
|  | What factors used to determine end of pounding process? (*possible to give multiple responses in order of importance*) | 1. Pounding duration 2. Number of pounding 3. Flatness(thickness/thinness) of pounded grains 4. Absence of un-dehusked grain 5. Other (specify)………………… |  |
|  | What is the pounding duration? (*specify time in minutes per specific amount pounded*) | ………………………………………………………………  ……………………………………………………………… |  |
|  | Does the pounding duration vary with given load? | 1. Yes 2. No   If yes, explain (how) ……………………………………….  ……………………………………………………………..  ………………......................................................................  ……………………………………………………………… |  |
|  | What is the method used to pound? (*possible to give multiple responses*) | 1. Manual by mortar and pestle 2. Mechanical/special machine 3. Other (specify) ……………. |  |
|  | What difficulties encountered during pounding? | ………………………………………………………………  ………………………………………………………………  ……………………………………………………………… |  |
|  | **Cleaning** (*remove of husk and other unwanted materials*) |  |  |
|  | Do you clean immediately after pounding? | 1. Yes 2. No |  |
|  | If yes, why? | ………………………………………………………………  ………………………………………………………………  ……………………………………………………………… |  |
|  | If not, how long do you store the product before cleaning, and why? | ………………………………………………………………  ………………………………………………………………  ……………………………………………………………… |  |
|  | Describe the place that you store them and the materials used? | ………………………………………………………………  ………………………………………………………………  ……………………………………………………………… |  |
|  | What are the common unwanted materials removed from the product? (*possible to give multiple responses*) | 1. Small broken grains/tips 2. Husks 3. Un-dehusked grains 4. Rice straws/leafs 5. Weed seeds 6. Puffed/popped grains 7. Other (specify)………………… |  |
|  | What is the method used to clean? (*possible to give multiple responses*) | 1. By hand (physical sorting) 2. Winnowing (traditional cleaning using wind) 3. Mechanical 4. Other (specify) ……………. |  |
|  | What difficulties encountered during cleaning? | ………………………………………………………………  ………………………………………………………………  ……………………………………………………………… |  |
|  | **Packaging and storage** *(Pepeta end product)* |  |  |
|  | How long (days/weeks) do you keep/store before selling the entire Pepeta product consignment? | ………………………………………………………………  ……………………………………………………………… |  |
|  | Under what conditions do you store Pepeta product? (*describe packaging materials, place, and equipments*) | ………………………………………………………………  ………………………………………………………………  ………………………………………………………………  ……………………………………………………………… |  |
|  | How long (days/weeks/months) is the shelf life of Pepeta? | …………………………………………. |  |
|  | What determine the end of shelf life (quality defects) of Pepeta products and why? | ………………………………………………………………………………………………………………………………………………………………………………………... |  |
|  | What difficulties encountered during packaging and storage? | ………………………………………………………………  ………………………………………………………………  ……………………………………………………………… |  |
|  | What is the most tedious production process/unit operation (in entire Pepeta processing) and why? | ………………………………………………………………  …………………………………………………………………………………………………………………………… |  |
|  | What is the number and gender of help people (if any) for entire Pepeta processing process at a time? (*mention the cost, in case of hired labour*) | 1. Female: ……………. 2. Male: ………………. |  |
|  | Can you give suggestions for improving the processing process? (*processing step optimisation, use of mature dried paddy etc*) | ……………………………………………………………..  ……………………………………………………………..  ……………………………………………………………..  …………………………………………………………….. |  |

**Section 5**: **Pepeta product trading**

| **N°** | **Questions** | **Answers** | **Code** |
| --- | --- | --- | --- |
|  | What is the main use(s) of Pepeta? | 1. Home consumption 2. Sale 3. Both |  |
|  | Does Pepeta price vary according to its freshness | 1. Yes 2. No |  |
|  | If yes, how much do you sell 1 kg of Pepeta (TZS)? (*estimate in kg if sold in volume*) | 1. Freshly prepared Pepeta product….………………… 2. Old prepared Pepeta product…………………………. |  |
|  | Does Pepeta price vary according to the period/season? | 1. Yes 2. No |  |
|  | If yes, in which period is the price low? | ………………………………………………… |  |
|  | How much do you sell one kilogram of Pepeta (TZS)? (*estimate in kg if sold in volume*) | 1. High price season….……………………… 2. Low price season.…………………………. |  |
|  | How much of the Pepeta (kg) do you sell per week/month? | …………………………………… |  |
|  | Where do you sell Pepeta?  (*possible to give multiple responses in order of importance*) | 1. Local market 2. Road side selling centre 3. Mobile call outs 4. Middle persons 5. Company/supermarket 6. Cooperatives 7. Others (specify)…………. |  |
|  | If you have some specific customers (*middle persons, company/ supermarket, cooperative*), could you give us the contact details (address & phone number) | 1. Address …………………………….   ………………………………………   1. Phone number………………………………….. |  |
|  | What are the customers preferences related to the quality of the Pepeta product, and what the most preferred one?  (*aroma, colour, appearance, hardness/softness, thickness/ thinness, etc*) | ……………………………………………………………….  ……………………………………………………………….  ……………………………………………………………….  ……………………………………………………………….  ……………………………………………………………… |  |
|  | Do you think that your customers are satisfied by the quality of the Pepeta purchased? | 1. Yes 2. No |  |
|  | If yes, explain? | ……………………………………………………………….  ……………………………………………………………….  ………………………………………………………………. |  |
|  | If not, why? | ……………………………………………………………….  ……………………………………………………………….  ………………………………………………………………. |  |
|  | What are the different uses of Pepeta that you know? (*more than one choice is acceptable*) | 1. Breakfast (main meal 2. Lunch (main meal) 3. Super (main meal) 4. Snack (before or after main meals) 5. Others (specify) ……….. |  |
|  | If consumed as snack, at what particular events and why? | ……………………………………………………………….  ……………………………………………………………….  ………………………………………………………………. |  |
|  | Do you still have anything to add to what we just discussed? | 1. Yes 2. No   If yes, explain ……………………………………………….  ……………………………………………………………….  ………………………………………………………………. |  |
|  | Do you perhaps have a question to me before we end our interview? | 1. Yes 2. No   If yes, write it…………. ……………………………………  ……………………………………………………………….  ………………………………………………………………. |  |

**Part III: Questionnaire for Pepeta product sellers**

**Section 6:** **Pepeta purchased for selling**

| **N°** | **Questions** | **Answers** | **Code** |
| --- | --- | --- | --- |
|  | Where do you buy Pepeta for selling? | 1. Buy from the market /sales point 2. From the processors at home 3. Other (specify)….…………………. |  |
|  | What parameters do you consider when buying Pepeta? (*possible to give multiple responses* ) | 1. Appearance 2. Color (greenness/whiteness) 3. Aroma 4. Taste 5. Texture (hardness/softness) 6. Other (specify)…………………… |  |
|  | For selected characteristics above, why them and what is the most important? | ……………………………………………........................................  …………………………………………………………..…………..  ……………………………………………………………………....  ……………………………………………………………………....  ……………………………………………………………………… |  |
|  | What are reasons to reject Pepeta product? | ……………………………………………………………………....  ………………………………………………………………………  ………………………………………………………………………  ………………………………………………………………………  ……………………………………………………………………… |  |
|  | How often do you purchase Pepeta product? | 1. More than 1 time /week 2. 1/week 3. 2/month 4. 1/month 5. Other (specify): |  |
|  | How much Pepeta do you buy a week/month? (*estimate in kg*) | …………………………………………………. |  |
|  | In which period is Pepeta purchase low? | …………………………………………………. |  |
|  | In which period is Pepeta purchase high? | …………………………………………………. |  |
|  | In what form is Pepeta packed when purchased? (*mention packaging materials/container*) | ……………………………………………………..  …………………………………………………….. |  |
|  | Are you satisfied by the quality of Pepeta purchased? | 1. Yes 2. No |  |
|  | If yes, explain? | ………………………………………………………………………  ……………………………………………………………………… |  |
|  | If not, why? | ………………………………………………………………………  ……………………………………………………………………… |  |
|  | How long (days/weeks) do you keep/store before selling the entire Pepeta product consignment? | ……………………………………………………………… |  |
|  | Under what conditions do you store Pepeta product? (*describe packaging materials, place, and equipments*) | ………………………………………………………………............  ………………………………………………………………………  ………………………………………………………………………  ………………………………………………………………………  ……………………………………………………………………… |  |
|  | How long (days/weeks/ months) is the shelf life of Pepeta? | ……………………………………………………………………… |  |
|  | What determine the end of shelf life (quality defects) of Pepeta products and why? | ………………………………………………………………………  ………………………………………………………………………  ………………………………………………………………………  ……………………………………………………………………… |  |

**Section 7**: **Pepeta trading**

| **N°** | **Questions** | **Answers** | **Code** |
| --- | --- | --- | --- |
|  | Does Pepeta price vary according to its freshness | 1. Yes 2. No |  |
|  | If yes, how much do you sell one kilogram of Pepeta (TZS)? (*estimate in kg if sold in volume*) | 1. Freshly prepared Pepeta product….………………… 2. Old prepared Pepeta product…………………………. |  |
|  | Does Pepeta price vary according to the period/season? | 1. Yes 2. No |  |
|  | How much do you sell one kilogram of Pepeta (TZS)? (*estimate in kg if sold in volume*) | 1. High price season….……from …...…… to................. 2. Low price season….…… from …...…… to................. 3. General price (if does not vary) ................................ |  |
|  | Where do you sell Pepeta product? | …………………………………………………………………  ………………………………………………………………… |  |
|  | Do you have specific customers? | 1. Yes 2. No |  |
|  | If yes, who are they? (*possible to give multiple responses)* | 1. Individuals 2. Association 3. Company/supermarket 4. Other (specify)…………………….. |  |
|  | If company/supermarket/ association could you give us the address? | 1. Address ………………………………………………….   …………………………………………………………..   1. Phone number…………………………… |  |
|  | What are the customers preferences related to the quality of the Pepeta product, and what the most preferred one?  (*aroma, colour, appearance, hardness/softness, thickness/ thinness,taste,oldor etc*) | ……………………………………………………………………  ……………………………………………………………………  ……………………………………………………………………  ……………………………………………………………………  ……………………………………………………………………  ……………………………………………………………………  …………………………………………………………………… |  |
|  | Do you think that your customers are satisfied by the quality of the Pepeta purchased? | 1. Yes 2. No |  |
|  | If yes, explain? | ……………………………………………………………………  …………………………………………………………………… |  |
|  | If not, why? | ……………………………………………………………………  …………………………………………………………………… |  |
|  | What are the different uses of the product that you know? | 1. Breakfast 2. Snack (before or after main meals) 3. Other use (specify)……………… |  |
|  | What challenges exist in Pepeta business? | ……………………………………………………………………  ……………………………………………………………………  …………………………………………………………………… |  |
|  | What are your suggestions for identified challenges above? | ……………………………………………………………………  ……………………………………………………………………  …………………………………………………………………… |  |
|  | Do you still have anything to add to what we just discussed? | 1. Yes 2. No   If yes, explain ……………………………………………………  …………………………………………………………………… |  |
|  | Do you perhaps have a question to me before we end our interview? | 1. Yes 2. No   If yes, write it…………. ………………………………………...  ………………………………………………………………….... |  |

**Part IV: Questionnaire for Pepeta consumers**

**Section 8:** **Pepeta purchased**

| **N°** | **Questions** | **Answers** | **Code** |
| --- | --- | --- | --- |
|  | How do you obtain Pepeta product? (*more than one choice is acceptable*) | 1. Own processing 2. Buy from the market/supermarket 3. From the processors at home 4. Other (specify)……………… |  |
|  | What characteristics do you consider when you buy/consume Pepeta? (*possible to give multiple responses* ) | 1. Appearance 2. Color (greenness/whiteness) 3. Aroma 4. Taste 5. Texture (hardness/softness) 6. Other (specify)…………………… |  |
|  | For selected characteristics above, why them and what is the most important? | ……………………………………………...........................  ……………………………………………………………...  ……………………………………………………………... |  |
|  | What are reasons to reject Pepeta product? | ……………………………………………...........................  ……………………………………………………………... |  |
|  | What is the frequency of Pepeta purchase? | 1. Once/month 2. Twice/month 3. More than twice/month (specify).......................... |  |
|  | How much of Pepeta do you purchase a week/month? (*estimate in kg* ) | …………………………………… |  |
|  | Does Pepeta price vary according to its freshness | 1. Yes 2. No |  |
|  | If yes, how much do you buy 1 kg of Pepeta (TZS)? (*estimate in kg if sold in volume*) | 1. Freshly prepared Pepeta product….………………… 2. Old prepared Pepeta product…………………………. |  |
|  | Does Pepeta price vary according to the period/season? | 1. Yes 2. No |  |
|  | How much do you purchase one kilogram of Pepeta (TZS)? (*estimate in kg if sold in volume*) | 1. High price season….……from …...…… to................. 2. Low price season….…… from …...…… to................. 3. General price (if does not vary) ................................ |  |
|  | In what form is Pepeta packed when purchased? (*mention packaging materials/container*) | ……………………………………………………………...  ……………………………………………………………...  ……………………………………………………………... |  |
|  | Are you satisfied by the quality of Pepeta purchased? | 1. Yes 2. No |  |
|  | If yes, explain? | ……………………………………………………………...  ……………………………………………………………... |  |
|  | If not, why? | ……………………………………………………………...  ……………………………………………………………... |  |
|  | What challenges exist in accessing Pepeta product? | ………………………………………………………………  ……………………………………………………………… |  |

**Section 9: Pepeta consumed**

| **N°** | **Questions** | **Answers** | **Code** |
| --- | --- | --- | --- |
|  | How long (days/weeks) do you keep/store before consume the entire purchased Pepeta product consignment? | …………………………………………………………. |  |
|  | What is the frequency of Pepeta consumption? | 1. Once a day 2. Twice a day 3. More than twice a day (specify)........ 4. Other (specify)…..…… |  |
|  | How many (household members) do you consume the entire Pepeta amount purchased in 2.6 above? | ……………………………………………….. |  |
|  | Under what conditions do you store Pepeta product? (*describe packaging materials, place, and equipments*) | ………………………………………………………………  …………………………………………………....................  ……………………………………………………………… |  |
|  | How long (days/weeks/ months) is the shelf life of Pepeta? | ……………………………………………………………… |  |
|  | What determine the end of shelf life (quality defects) of Pepeta products and why? | ………………………………………………………………  ………………………………………………………………  ……………………………………………………………… |  |
|  | In which period/season do you consume Pepeta? | ………………………………………………………………  ……………………………………………………………… |  |
|  | How do you consume Pepeta product?  (*more than one choice is acceptable*) | 1. Breakfast (main meal 2. Lunch (main meal) 3. Supper (main meal) 4. Snack (before or after main meals) 5. Others............ |  |
|  | If consumed as snack, in what particular events and why? | ……………………………………………………………….  ………………………………………………………………. |  |
|  | Can you give suggestions for improving Pepeta production/accessibility? | ………………………………………………………………  ………………………………………………………………  ……………………………………………………………… |  |
|  | Do you still have anything to add to what we just discussed? | 1. Yes 2. No   If yes, explain ……………………………………………….  ………………………………………………………………. |  |
|  | Do you perhaps have a question to me before we end our interview? | 1. Yes 2. No   If yes, write it…………. ……………………………………  ……………………………………………………………….  ……………………………………………………………….  ………………………………………………………………. |  |

**End of questionnaire**

**Thank you**
