# Supplementary material for: Utilization of Pepeta, a locally processed immature rice-based food product, to promote food security in Tanzania
Source: PLoS One. 2021 Mar 3;16(3):e0247870. doi: 10.1371/journal.pone.0247870 (PMC7928465; doi:10.1371/journal.pone.0247870)
Supplement: S1 Checklist — (DOCX) [file pone.0247870.s002.docx]

**S1 Checklist. Indigenous processing knowledge and consumers’ preferences on *pepeta***

**GPS coordinates:**

_______________________

_______________________

**Checklist number**

**|___|___|___|___|___|___|**

**|District | Ward | Village |**

1. Introduction

*Group name, location, date, number of participants (Male Vs female, farmers vs processors, age distribution)*

---------------------------------------------------------------------------------------------------------------------------------------------------------------------------------------------------------------------------------------------------------------------------------------------------------------------------------------

1. Background

*What common rice varieties currently cultivated (landraces & improved/hybrid) & why?*

---------------------------------------------------------------------------------------------------------------------------------------------------------------------------------------------------------------------------------------------------------------------------------------------------------------------------------------------------------------------------------------------------------------------------------------------------------------------------------------------------------------------------------------------------------------

1. Communities’ perceptions and knowledge on rice quality and rice –based food products
   1. *Indicators of good rice variety and why?*

--------------------------------------------------------------------------------------------------------------------------------------------------------------------------------------------------------------------------------------------------------------------------------------------------------------------------------------------------------------------------------------------------------------------------------------------------------------------------------------------------------------------------------------------------------------------------------------------------------------------------------------------------------

- 1. *What are common rice-based food products in this locality? (Pepeta, Polished/white rice, Brown rice, Parboiled rice, Mchopeko/ traditional parboiled rice, Vitumbua / rice dough, Mkate wa kumimina /rice bread, Unga wa lishe / composite flour, Visheti /biscuits)*

--------------------------------------------------------------------------------------------------------------------------------------------------------------------------------------------------------------------------------------------------------------------------------------------------------------------------------------------------------------------------------------------------------------------------------------

1. Pepeta product
   1. Pepeta background and uses *(how was it discovered, different uses associated with particular event, common varieties used, most used variety & its characteristics – appearance, color, aroma, size etc., Pepeta quality factors & the most preferred one)*

---------------------------------------------------------------------------------------------------------------------------------------------------------------------------------------------------------------------------------------------------------------------------------------------------------------------------------------

- 1. Pepeta processing
     1. How is cutting done? *(maturity identification for Pepeta processing, maturity level, days the paddy in the field still suitable for Pepeta from first harvest etc.)*

---------------------------------------------------------------------------------------------------------------------------------------------------------------------------------------------------------------------------------------------------------------------------------------------------------------------------------------

- - 1. How is threshing done? *(method and equipment used, difficulties encountered etc.)*

---------------------------------------------------------------------------------------------------------------------------------------------------------------------------------------------------------------------------------------------------------------------------------------------------------------------------------------------------------------------------------------------------------------------------------------------------

- - 1. How is cleaning/sorting done? *(method and equipment used, unwanted materials removed, difficulties encountered etc.)*

---------------------------------------------------------------------------------------------------------------------------------------------------------------------------------------------------------------------------------------------------------------------------------------------------------------------------------------

- - 1. How is roasting done? *(time interval between sorting & roasting, factors used to determine end of roasting process, roasting duration & factors affecting it, method used, energy source & its availability, difficulties encountered etc.)*

---------------------------------------------------------------------------------------------------------------------------------------------------------------------------------------------------------------------------------------------------------------------------------------------------------------------------------------------------------------------------------------------------------------------------------------------------

- - 1. How is pounding done? *(time interval between roasting & pounding, factors used to determine end of pounding process, pounding duration & factors affecting it, method used, difficulties encountered etc.)*

--------------------------------------------------------------------------------------------------------------------------------------------------------------------------------------------------------------------------------------------------------------------------------------------------------------------------------------------------------------------------------------------------------------------------------------------------

- - 1. How is cleaning done? *(time interval between pounding & cleaning, common unwanted materials removed, method used, difficulties encountered etc.)*

---------------------------------------------------------------------------------------------------------------------------------------------------------------------------------------------------------------------------------------------------------------------------------------------------------------------------------------------------------------------------------------------------------------------------------------------------

- - 1. Packaging and storage *(storage conditions, Pepeta shelf life and factors determine its end, difficulties encountered etc.)*

---------------------------------------------------------------------------------------------------------------------------------------------------------------------------------------------------------------------------------------------------------------------------------------------------------------------------------------

- 1. What are the most tedious production process/unit operations (in entire Pepeta processing) and why?

---------------------------------------------------------------------------------------------------------------------------------------------------------------------------------------------------------------------------------------------------------------------------------------------------------------------------------------

- 1. What are your suggestions for improving the processing process? (*processing step optimisation, use of mature dried paddy etc.)*

-------------------------------------------------------------------------------------------------------------------------------------------------------------------------------------------------------------------------------------------------------------------------------------------------------------------------------------------------------------------------------------------------------------------------------------------------------------------------------------------------------------------------------------------------------------------------------------------------------------------------------------------------------------------------------------------------------------------------------------------------------------------------------------

**Thank you**
